# Exosomes: innovative biomarkers leading the charge in non-invasive cancer diagnostics

**DOI:** 10.7150/thno.113650

**Published:** 2025-04-13

**Authors:** Jiale Li, Ailin Wang, Haijun Guo, Wei Zheng, Rui Chen, Changfeng Miao, Dandan Zheng, Jun Peng, Jiachong Wang, Zigui Chen

**Affiliations:** 1 Department of Neurosurgery, Haikou Affiliated Hospital of Central South University Xiangya School of Medicine, Haikou, China, 570208.; 2 School of Basic Medicine and Clinical Pharmacy, China Pharmaceutical University, Nanjing, China, 211198.; 3 Department of Neurosurgery, Central Hospital of Zhuzhou, Zhuzhou, Hunan, China, 412000.; 4 Department of Neurosurgery, Affiliated Nanhua Hospital, University of South China, Hengyang, Hunan, China, 533000.; 5 Department of Neurosurgery Second Branche, Hunan Provincial People's Hospital (The First Affiliated Hospital of Hunan Normal University), Changsha, Hunan, China, 410005.; 6 Department of Radiation Oncology, The First Affiliated Hospital Zhejiang University, Hangzhou, China, 310009.

## Abstract

Exosomes, nanoscale extracellular vesicles secreted by diverse cell types, have emerged as promising biomarkers for non-invasive tumor diagnostics, offering significant advantages over traditional methods. These vesicles, typically ranging from 30 to 150 nanometers in size, carry a diverse cargo of proteins, lipids, RNA, and microRNAs, which reflect the molecular alterations occurring within their parent cells. Notably, exosomes can be isolated from easily accessible biofluids such as blood, urine, and saliva, making them ideal candidates for liquid biopsy applications. This review explores the transformative potential of exosome-based biomarkers in the early detection and monitoring of cancers across diverse organ systems, including respiratory, digestive, hematological, neurological, endocrine malignancies and so on. Special emphasis is placed on their application in clinical trials, where exosome-based diagnostics have demonstrated promising results in detecting tumors at early stages and monitoring treatment responses, offering a less invasive and more accessible alternative to traditional biopsies. While recent advancements in exosome isolation and characterization technologies have significantly improved the sensitivity and specificity of these diagnostics, challenges such as biological heterogeneity, lack of standardization, and regulatory hurdles remain. Nevertheless, exosome-based diagnostics hold the promise of providing real-time, dynamic insights into tumor progression, enhancing personalized medicine. The integration of exosomes into clinical practice could revolutionize cancer diagnostics and therapy, improving patient outcomes. Further research and large-scale clinical validation are essential to fully realize the clinical potential of exosome-based biomarker applications in routine clinical settings.

## 1. Introduction

**Figure 1:**
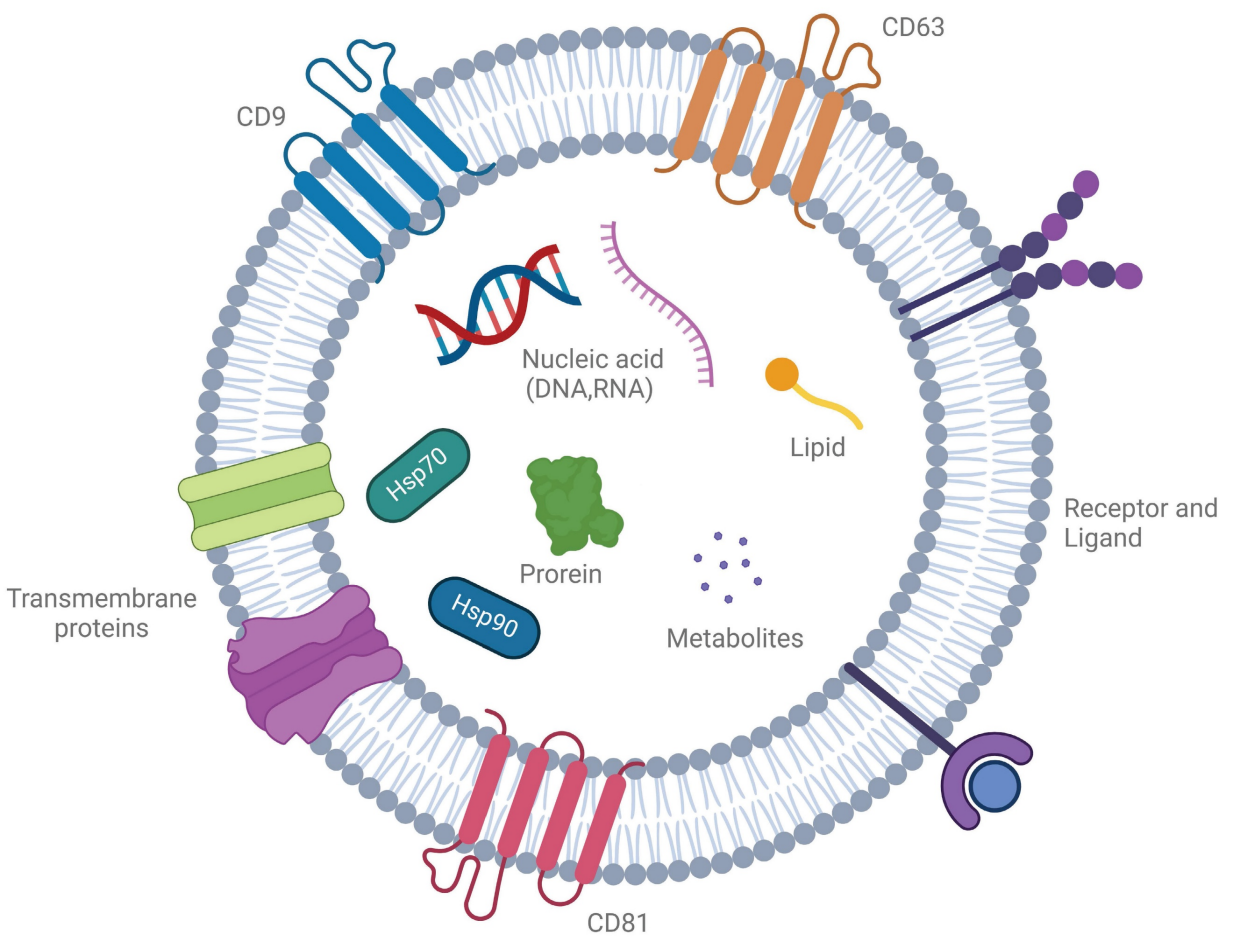
A cell-to-cell transit system in the human body with pleiotropic functions. Exosomes are extracellular vesicles generated by all cells and they carry nucleic acids, proteins, lipids, and metabolites. They are mediators of near and long-distance intercellular communication in health and disease and affect various aspects of cell biology. Created with BioRender.com.

Exosomes are nanosized, membrane-bound vesicles ranging from 30 to 150 nm, produced by many cell types, including cancer cells, immune cells, and neurons. These vesicles play a crucial role in intercellular communication by transferring bioactive molecules such as proteins, lipids, RNAs, and metabolites 1 (Figure 1). Known for protecting their cargo from degradation, exosomes are key messengers in both normal and disease-related processes, reflecting the molecular profile of their parent cells. Particularly, exosomes derived from diseased cells, such as cancer or infected cells, carry disease-specific signatures, making them promising candidates for non-invasive diagnostic applications 2.

Traditionally, disease diagnosis has relied on invasive techniques like tissue biopsies. Although these are valuable, they are limited by sampling biases, the need for complex surgical procedures, and the inability to capture the dynamic progression of diseases. This is especially true for cancers, where the molecular variation across tumor regions complicates diagnostic accuracy 3. As an alternative, liquid biopsy--a non-invasive approach analyzing biofluids such as blood, urine, and saliva for disease-related biomarkers--has gained attention. Exosomes, due to their stable presence in these biofluids, have become ideal candidates for liquid biopsy. Their molecular content mirrors the alterations in disease states, offering real-time insights into disease detection, monitoring, and progression 4. Recent advancements in exosome isolation and characterization techniques, such as ultracentrifugation, immunoaffinity capture, and microfluidics, have further boosted the potential of exosome-based diagnostics 5. These technologies allow for the extraction and analysis of exosomal proteins, RNAs, and lipids, which have shown promise as biomarkers for early disease detection and monitoring. For example, exosomal miRNAs and proteins have been identified as biomarkers for early-stage cancers, while exosomal RNAs have been used to detect infectious diseases and monitor neurodegenerative conditions 6.

However, several challenges must be addressed to fully realize the diagnostic potential of exosomes. These include the need for standardized, scalable isolation methods, the identification of specific and sensitive biomarkers, and the clinical validation of exosome-based diagnostics across diverse patient populations. Additionally, the heterogeneity of exosomes, both within and between diseases, presents another challenge for their widespread use 7. This review aims to provide a comprehensive overview of exosomal biomarkers and their transformative potential in non-invasive diagnostics. By examining the current state of exosome-based diagnostic technologies, the molecular potential of exosomal content, and the clinical feasibility of their application, we seek to highlight impact of exosomes on revolutionizing early disease detection, monitoring disease progression, and facilitating personalized medicine. Ultimately, the integration of exosome-based diagnostics into clinical practice holds the promise of improving patient outcomes by offering a more precise, less invasive, and accessible approach to disease management.

## 2. Exosome biogenesis and role in tumors

### 2.1. Biogenesis of exosomes

**Figure 2:**
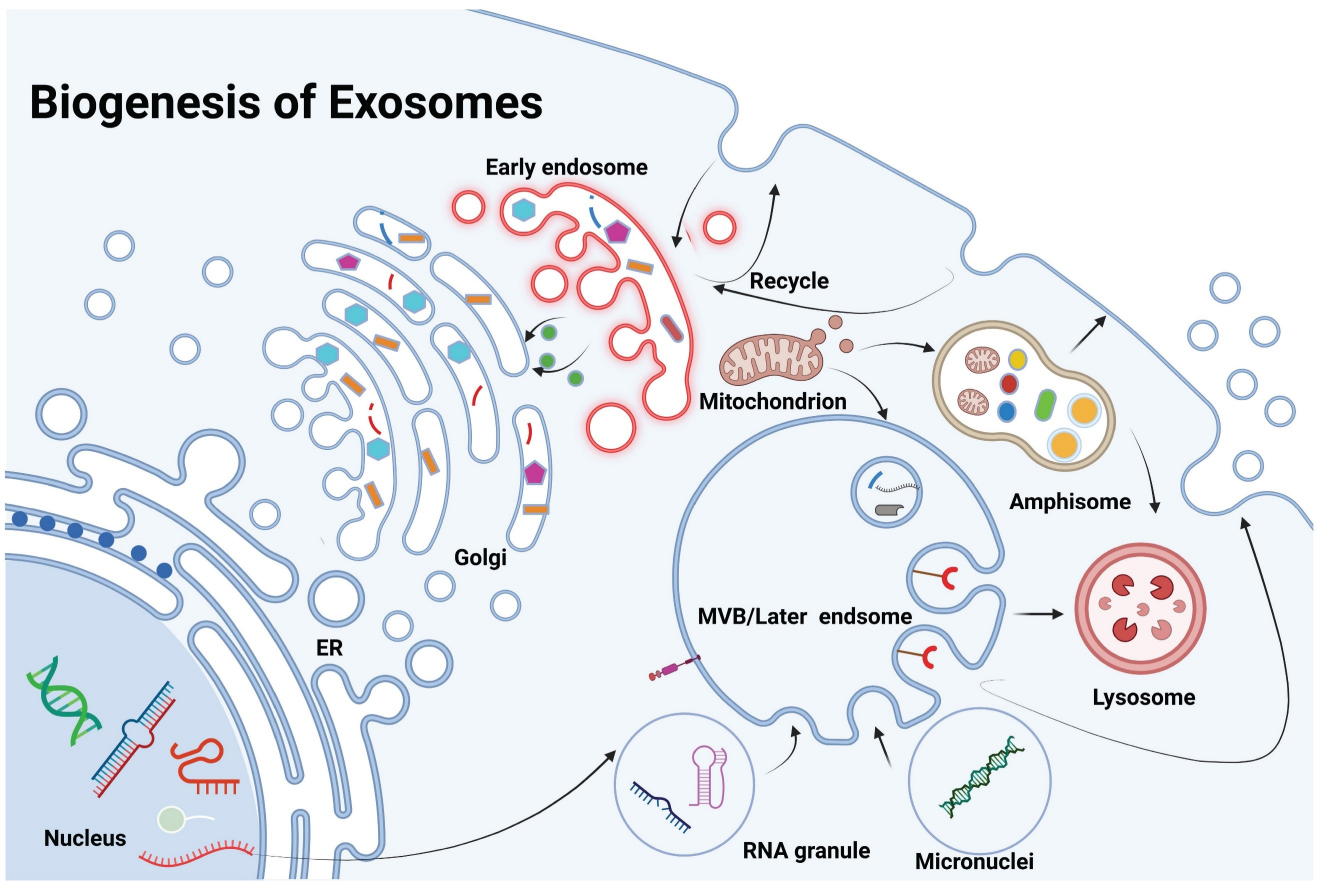
Overview of the process for exosome biogenesis. Exosome biogenesis is primarily centered around the formation of multivesicular bodies (MVBs). These structures are typically derived from the process of endocytosis, during which various mechanisms facilitate the inward budding of the plasma membrane, leading to the creation of early endosomes. As these endosomes mature, they undergo further processes that result in the formation of MVBs, which ultimately give rise to exosomes. Created with BioRender.com.

Exosome biogenesis is a highly regulated, multi-step process involving the formation, maturation, and release of multivesicular bodies (MVBs), which are key intermediates in exosome production. This process begins with the invagination of the plasma membrane to form early endosomes that sort cellular cargo. As these early endosomes mature into late endosomes or MVBs, further inward budding generates intraluminal vesicles (ILVs), which become exosomes upon release. These ILVs selectively encapsulate lipids, proteins, and nucleic acids, reflecting the physiological or pathological state of the source cell 8 (Figure 2). The release of exosomes is regulated by the fate of MVBs, which may either fuse with lysosomes for degradation or with the plasma membrane to release exosomes. This process is controlled by Rab GTPases, such as Rab27a and Rab27b, ensuring proper timing and spatial regulation of exosome release 9. There are two primary pathways for ILV formation and cargo sorting: the ESCRT-dependent and ESCRT-independent pathways. The ESCRT-dependent pathway involves the endosomal sorting complex required for transport (ESCRT), which mediates the formation of ILVs through a series of sequential complexes (ESCRT-0, -I, -II, and -III). ESCRT-0 recognizes ubiquitinated proteins, while ESCRT-I and -II facilitate membrane deformation, and ESCRT-III completes vesicle scission. Accessory proteins like Alix and TSG101 assist in this process, with VPS4 providing energy for complex disassembly. The ESCRT-independent pathway relies on lipid and protein-based mechanisms. Ceramide, generated by neutral sphingomyelinase 2 (nSMase2), induces membrane curvature, and tetraspanins like CD9, CD63, and CD81 organize the membrane and aid in cargo sorting 10. Cargo loading into ILVs is highly selective and involves various molecular players. Heat shock proteins (e.g., HSP90, HSP70) incorporate functional proteins, while RNA-binding proteins like hnRNPA2B1 and YBX1 selectively package microRNAs and other non-coding RNAs into exosomes. Lipids such as cholesterol and phosphatidylserine stabilize the vesicles and facilitate membrane fusion during release 11.

The above-explained mechanisms of exosome biogenesis are closely linked to tumor initiation and progression. In cancer, dysregulated exosome biogenesis leads to the secretion of exosomes that carry oncogenic cargo, influencing the tumor microenvironment (TME) and promoting tumor progression. These exosomes, enriched with growth factors, metalloproteinases, and microRNAs, reprogram normal cells into malignant phenotypes and contribute to clonal expansion and intratumoral heterogeneity. Exosomes also facilitate immune evasion by carrying immunosuppressive molecules such as PD-L1, TGF-β, and IL-10, while promoting metastasis through the transfer of matrix metalloproteinases (MMPs). Furthermore, exosomes play a key role in drug resistance by transferring resistance factors, including drug efflux pumps and mutant proteins, along with miRNAs that affect apoptosis and drug metabolism, complicating treatment strategies 12.

The dynamic interplay between exosome biogenesis and cancer progression highlights the importance of exosomes as not only vehicles for intercellular communication but also active mediators of tumor progression, immune modulation, and treatment resistance. Given their central role in these processes, exosomes present an intriguing therapeutic target for modulating cancer progression, as well as a non-invasive biomarker for early detection, monitoring disease progression, and evaluating therapeutic responses. Understanding the precise molecular mechanisms underlying exosome biogenesis and cargo selection in cancer cells is essential for the development of exosome-based therapies and diagnostic tools 13.

### 2.2. The role of exosomes in tumor progression

**Figure 3:**
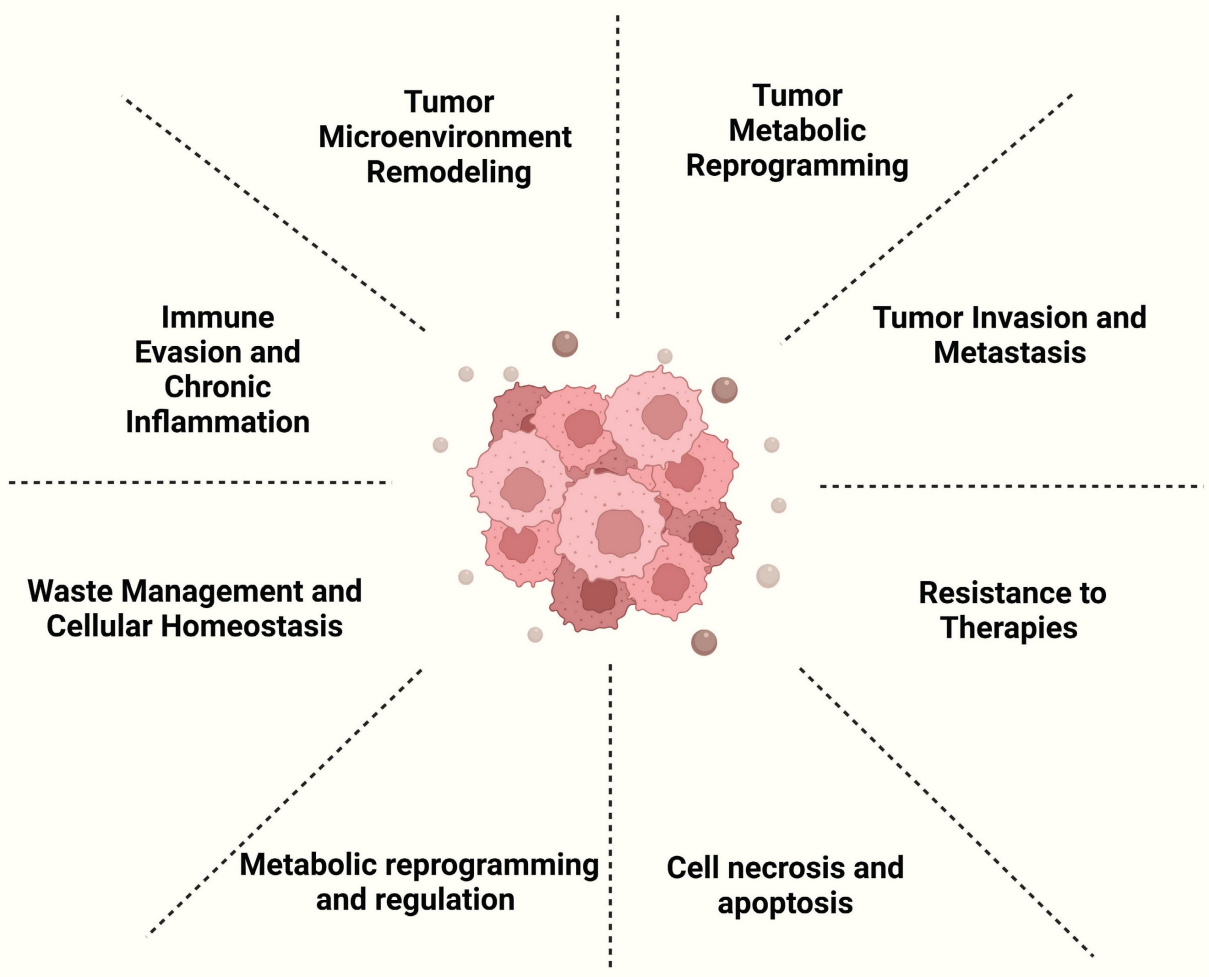
The role of exosomes in tumor progression. Created with BioRender.com.

Exosomes serve as a key factor in cancer progression by remodeling the tumor microenvironment and influencing key processes such as tumor cell growth, migration, immune evasion, and therapy resistance. They facilitate tumor growth by transporting molecules, including non-coding RNAs, that regulate cancer cell behavior and promote angiogenesis. Exosomes also trigger macrophage polarization and chronic inflammation, enhancing tumor survival and dissemination. Furthermore, exosomes have been identified as key mediators of resistance to cancer therapies, as they carry molecules associated with drug resistance, diminishing the efficacy of therapies including chemotherapy and radiotherapy. Due to their presence in body fluids, exosomes hold significant potential as non-invasive biomarkers for cancer detection and prognosis 14 (Figure 3). Below, we provide a selection of representative examples that, while not exhaustive, highlight their significance.

#### 2.2.1. Tumor Microenvironment (TME) remodeling

Exosomes are pivotal mediators of intercellular communication within the TME, a sophisticated ecosystem consisting of tumor cells, stromal cells (including cancer-associated fibroblasts or CAFs), immune cells, blood vessels, and the extracellular matrix (ECM) 15. These nanoscale vesicles, containing bioactive molecules such as proteins, RNAs (including miRNAs, mRNAs, and lncRNAs), lipids, and metabolites, profoundly influence the TME's dynamics, facilitating tumor progression, metastasis, and immune evasion. Tumor-derived exosomes alter the behavior of surrounding non-tumor cells by transferring tumor-specific molecules, such as oncogenic miRNAs, which induce CAFs to adopt pro-tumor phenotypes, thereby promoting tumor growth, invasion, and metastatic potential 16. Additionally, exosomes facilitate immune evasion by delivering immunosuppressive molecules, including PD-L1 and TGF-β, which dampen anti-tumor immune responses and reprogram immune cells, including macrophages, towards an immunosuppressive M2 phenotype. Furthermore, exosomes are crucial for tumor angiogenesis by transferring angiogenic factors, such as VEGF, to endothelial cells, stimulating new blood vessel formation and increasing endothelial barrier permeability. This facilitates tumor cell extravasation and metastasis. Exosomes also mediate ECM remodeling by carrying matrix metalloproteinases (MMPs) and other proteases that degrade ECM components, including fibronectin and collagen, thereby enhancing tumor cell invasion and facilitating metastatic spread. Through these mechanisms, exosomes serve as critical modulators of the TME, promoting tumor progression, immune escape, and metastasis, and therefore represent promising targets for therapeutic intervention 17.

#### 2.2.2. Tumor metabolic reprogramming

Tumor cells undergo significant metabolic reprogramming, which is considered one of the hallmarks of cancer. This adaptation enables them to meet the increased energy demands required for rapid proliferation and survival in hostile microenvironments. A hallmark of this metabolic shift is the Warburg effect, where cancer cells mainly depend on glycolysis for ATP generation, even under normoxic conditions. This metabolic reprogramming facilitates cancer cells to efficiently produce energy and biosynthetic essential intermediates for growth. In addition to glucose metabolism, altered lipid and amino acid metabolism are also essential for sustaining cancer cell growth and metastasis 18. Exosomes, small vesicles secreted by tumor cells, serve as key mediators of metabolic reprogramming within the TME. These extracellular vesicles carry a diverse array of molecules that allow exosomes to facilitate communication between surrounding stromal cells and cancer cells. Exosomes from tumor cells transfer metabolic signals, such as lactate, fatty acids, and miRNAs, to neighboring cells, including CAFs, endothelial cells, and immune cells, thereby promoting the metabolic adaptation necessary for tumor growth 19. One of the central processes driven by exosomes is the "reverse Warburg effect." Tumor cells export lactate via exosomes, which is then taken up by CAFs. This lactate is converted back into pyruvate, which fuels oxidative phosphorylation (OXPHOS) in CAFs, providing them with the energy needed to support tumor cells. This metabolic symbiosis between cancer cells and stromal cells facilitates the continuous growth and invasion of tumors. Additionally, exosomes enable metabolic reprogramming of immune cells within the TME. For instance, exosome-mediated transfer of metabolic molecules can alter the function of macrophages, promoting their polarization toward an immunosuppressive M2 phenotype that supports tumor progression and immune evasion 20. Exosomes are also key contributors to the regulation of amino acid and lipid metabolism within the TME. Exosome-mediated transfer of essential metabolic intermediates enhances tumor cell proliferation and survival. By reprogramming the metabolic landscape of stromal and immune cells, exosomes create a supportive environment that favors tumor progression, metastasis, and resistance to therapeutic interventions 21.

#### 2.2.3. Immune evasion

Immune evasion is a fundamental mechanism that enables tumor cells to escape recognition and destruction by the host immune system, contributing significantly to cancer progression, metastasis, and therapeutic resistance. Tumors exploit a variety of strategies to subvert immune surveillance, ensuring their survival and growth within the hostile TME. These mechanisms include immune suppression, alteration of immune cell function, and modulation of immune checkpoints, which collectively enable tumor cells to evade both adaptive and innate immune responses 22. One of the primary immune evasion mechanisms is the reprogramming of tumor-associated immune cells, such as tumor-associated macrophages (TAMs), dendritic cells, and regulatory T cells (Tregs), into immunosuppressive phenotypes. For example, TAMs are frequently polarized toward an M2-like phenotype within the TME, promoting immune suppression and enhancing tumor progression. Tumor cells can also induce Tregs, which suppress the effectiveness of cytotoxic T cells and natural killer (NK) cells, further shielding the cancer from immune attack 23. Moreover, tumor cells frequently exploit immune checkpoint pathways, such as the PD-1/PD-L1 axis, to inhibit T cell activation. PD-L1 is often upregulated on the surface of tumor cells and immune cells within the TME, binding to the PD-1 receptor on T cells and suppressing their ability to mount an effective anti-tumor response. This mechanism not only prevents T cells from attacking the tumor but also promotes tumor cell survival by dampening the immune response 24. Other immune checkpoints, such as CTLA-4, are similarly exploited by tumors to impair immune activation and promote immune tolerance 25. Exosomes, small vesicles secreted by tumor cells, play a key role in immune evasion by transferring immunosuppressive molecules, including PD-L1, TGF-β, and immunomodulatory miRNAs, to immune cells. Through this mechanism, exosomes facilitate the polarization of macrophages into an M2 phenotype and promote Treg expansion, further suppressing anti-tumor immunity. Additionally, exosomes can transfer tumor antigens to dendritic cells, altering their function and impairing their ability to initiate a robust anti-tumor immune response 26. The metabolic reprogramming of the TME also contributes to immune evasion. Tumor cells often induce a hypoxic, acidic, and nutrient-deprived environment that inhibits the function of immune cells. For instance, low oxygen levels and high lactate concentrations within the TME can impair the cytotoxic activity of NK cells and CD8+ T cells, further facilitating immune escape. Tumor-derived exosomes, which carry metabolic molecules such as lactate, exacerbate this immunosuppressive microenvironment by influencing the metabolism of both immune and stromal cells 27.

#### 2.2.4. Tumor invasion and metastasis

Cancer progression through invasion and metastasis is a principal cause of morbidity and mortality, and the capacity of tumor cells to infiltrate local tissues and metastasize is essential for cancer progression 28. Exosomes, small vesicles secreted by tumor cells, act as a key factor in mediating the complex processes of invasion and metastasis. These nanoscale vesicles carry a range of bioactive molecules which facilitate communication between tumor cells and the surrounding TME, as well as distant organs involved in metastasis. Exosomes contribute to tumor invasion by promoting the degradation of the ECM, a crucial barrier that must be disrupted for tumor cells to invade surrounding tissues. Tumor-derived exosomes carry MMPs and urokinase-type plasminogen activator (uPA), both of which are enzymes that degrade ECM components such as collagen, fibronectin, and laminins. By facilitating ECM degradation, exosomes enable tumor cells to migrate and invade neighboring tissues, a critical step in the metastatic cascade 29. Furthermore, exosomes can modulate the phenotype of stromal cells, such as fibroblasts, endothelial cells, and immune cells, to create a permissive environment for invasion. For example, exosomes derived from tumor cells can induce CAFs to promote ECM remodeling and secretion of pro-inflammatory cytokines, which further support tumor cell invasion 30. In the context of metastasis, exosomes play a key role in establishing the pre-metastatic niche (PMN), a microenvironment in distant organs that is primed to support the survival and colonization of circulating tumor cells. Tumor-derived exosomes can transfer a variety of factors that alter the behavior of stromal cells, endothelial cells, and immune cells in distant organs, creating a favorable environment for metastasis 31. For instance, exosomes can recruit immune cells such as myeloid-derived suppressor cells (MDSCs) and macrophages to distant organs, where they facilitate immune suppression and support the metastatic process. Exosomes can also transfer factors such as vascular endothelial growth factor (VEGF), which promote angiogenesis and increase vascular permeability, allowing tumor cells to more easily extravasate from the bloodstream and colonize secondary tissues 32. Moreover, exosomes mediate signaling between tumor cells and distant metastatic sites by modulating the metabolic and immune landscape of the TME. Exosomes carry metabolic signals, including lactate and lipids, which influence the metabolism of stromal and immune cells in the metastatic microenvironment. These metabolic changes promote tumor cell survival and immune evasion at metastatic sites. Additionally, exosomes can modulate immune cell function by transferring immunosuppressive molecules such as PD-L1, TGF-β, and miRNAs, which help tumor cells evade immune detection and destruction in distant organs 33.

#### 2.2.5. Resistance to therapies

Drug-resistant tumors pose a significant therapeutic challenge in cancer treatment, contributing to therapy failure and poor patient outcomes 34. Exosomes, small vesicles secreted by tumor cells, are now pivotal in mediating the mechanisms underlying drug resistance 35. These extracellular vesicles carry a range of bioactive molecules that facilitate intercellular communication between tumor cells, stromal cells, and immune cells within the TME. One of the primary mechanisms by which exosomes mediate drug resistance is through the transfer of molecules that modulate drug efflux. Tumor cells often upregulate ATP-binding cassette (ABC) transporters, such as P-glycoprotein, which actively expel chemotherapy drugs from the cell, causing reduced intracellular drug levels and compromised therapy outcomes 36. Exosomes play a critical role in this process by transferring these drug efflux pumps to neighboring cells, including CAFs and endothelial cells, thereby facilitating the spread of resistance across the tumor. This intercellular exchange of drug resistance markers contributes to the heterogeneous nature of resistance within a tumor, complicating treatment strategies 37. Exosomes also play a role in drug resistance by mediating changes in the tumor microenvironment that promote cellular survival and evade drug-induced apoptosis. For instance, exosomes can carry and transfer survival factors including epidermal growth factor receptor (EGFR) ligands, VEGF, and anti-apoptotic proteins like Bcl-2. By transferring these factors, exosomes enhance the proliferative and survival capabilities of recipient cells, making them less susceptible to chemotherapy and targeted therapies 38. Moreover, exosomes can carry miRNAs and lncRNAs that regulate apoptosis-related pathways, further contributing to the resistance phenotype. For example, exosome-mediated delivery of miR-21, which targets pro-apoptotic genes, has been demonstrated to enhance resistance to various chemotherapeutic agents 39. Additionally, exosomes can influence the immune response to cancer therapies, promoting immune evasion and resistance to immunotherapies. Tumor-derived exosomes often carry immunosuppressive molecules including PD-L1, TGF-β, and FasL, which can suppress the activity of cytotoxic T cells, NK cells, and other immune effector cells. By modulating the immune landscape of the TME, exosomes create an environment that supports tumor survival and enhances resistance to immune checkpoint inhibitors and other immunotherapies 40. The metabolic reprogramming of the TME also acts as a major factor in drug resistance, and exosomes are involved in this process. Tumor cells and their microenvironment often undergo metabolic changes, such as increased glycolysis, to support rapid growth and survival. Exosomes can carry metabolic molecules, including lactate and lipids, to neighboring cells, promoting a shift in the metabolic profile of the TME that supports drug resistance. For instance, exosome-mediated transfer of lactate to nearby cells has been shown to induce a metabolic shift that enables cells to resist oxidative stress and survive under low oxygen conditions, enhancing their resistance to chemotherapy and radiation therapy 41.

## 3. Conventional cancer diagnostic methods vs Exosome-based cancer diagnostics

### 3.1. Conventional cancer diagnostic methods

Conventional cancer diagnostic methods, including imaging techniques, histological examination, blood and biomarker testing, and molecular biological tests, are essential for early-stage detection, cancer staging, and prognosis evaluation. However, each method has limitations in sensitivity, specificity, and invasiveness 42. Imaging techniques, such as CT, MRI, PET, and ultrasound, provide critical information on tumor size, location, and metabolic activity, but may struggle with detecting early-stage cancer or distinguishing benign from malignant lesions. It is worth noting that, with the continuous advancement of diagnostics, the integration of radiomics and artificial intelligence, particularly PSMA-PET/CT imaging, can significantly enhance the diagnostic accuracy, staging, treatment planning, and outcome prediction for certain cancers 43. Histological examination offers reliable tumor classification and grading but lacks molecular insights. Blood-based tests, like liquid biopsies, offer non-invasive alternatives for detecting cancer-related biomarkers but can face challenges with false positives and negatives. Molecular biological tests, including NGS and PCR, provide detailed genetic insights, aiding in personalized treatment plans, but can be expensive and technically complex. Despite their advantages, these methods do not fully capture the tumor's molecular heterogeneity or microenvironment, limiting their ability to assess treatment responses and resistance 44.

### 3.2. Advantages of exosome-based diagnostics

Exosome biomarkers offer significant advantages over traditional diagnostic methods in cancer, providing a promising avenue for non-invasive, highly sensitive, and real-time monitoring of tumor dynamics. Exosomes are nanoscale extracellular vesicles secreted by tumor cells into the bloodstream and other bodily fluids, carrying a wealth of bioactive molecules, which reflect the genetic and phenotypic characteristics of the originating tumor. Unlike other biomarkers, exosomes are stable in circulation, making them reliable candidates for liquid biopsy applications, particularly for early cancer detection, monitoring treatment response, and detecting minimal residual disease 45. One of the primary benefits of exosome-based diagnostics is their ability to capture the heterogeneity of tumors. Tumors are often composed of genetically diverse cell populations, and exosomes provide a snapshot of this diversity by carrying molecular information from different tumor subclones. This feature allows exosomes to offer a more comprehensive understanding of tumor biology compared to traditional methods, which may fail to fully represent tumor heterogeneity. Moreover, exosomes can be derived from various biofluids, including blood, urine, and saliva, enabling minimally invasive sampling that provides an accessible and convenient alternative to tissue biopsy, which can be invasive and challenging to perform repeatedly 46. Exosomes also outperform conventional biomarkers in their ability to detect early-stage cancers and monitor real-time treatment response. Because exosomes contain tumor-specific molecules, their presence and composition change with tumor progression, offering valuable insights into disease status before the emergence of clinically detectable tumors. Additionally, exosome-derived biomarkers can be used to assess therapeutic efficacy, providing a dynamic picture of tumor evolution and resistance mechanisms. For example, the detection of exosome-mediated changes in protein expression or RNA levels can signal the development of drug resistance or immune evasion, facilitating early intervention and adjustment of therapeutic strategies 47. Furthermore, exosomes facilitate the detection of molecular signatures linked to immunotherapy, targeted therapy, and chemotherapy. Their ability to convey genetic, proteomic, and lipidomic information makes them powerful tools for personalized cancer care, facilitating the selection of the most appropriate treatment for individual patients. This molecular profiling capacity offers a level of precision that traditional biomarkers, such as serum protein markers, cannot match 48.

### 3.3. Limitations of exosome-based diagnostics

Exosome-based cancer diagnostics hold significant promise but face several challenges that hinder their clinical implementation. One major limitation lies in the purification and detection methods used to isolate and analyze exosomes. Common purification techniques, such as ultracentrifugation, size-exclusion chromatography (SEC), immunocapture, and precipitation kits, each come with their own advantages and limitations, especially in terms of yield, purity, and processing efficiency. These methods can produce exosome preparations of varying quality, which affects the consistency of biomarker identification 49. In addition, the sensitivity, specificity, and throughput of detection techniques can vary widely. Methods such as fluorescence-based techniques, nanoparticle tracking analysis (NTA), Western blotting, enzyme-linked immunosorbent assays (ELISA), and newer approaches like surface-enhanced Raman spectroscopy (SERS) and electrochemical sensors all offer unique capabilities, including label-free detection and multiplexing. However, these methods are not without their challenges, and their application depends on the clinical needs and the disease-specific characteristics of the exosomes. For instance, detecting low-abundance biomarkers in bodily fluids like blood requires highly sensitive techniques, which can be costly and time-consuming 50 (Table 1).

The inherent heterogeneity of exosomes further complicates their diagnostic application. Exosome content can differ significantly depending on the cancer type, tumor microenvironment, and even between individual patients, making it difficult to identify reliable, universal biomarkers for broad cancer detection. This variability, combined with a lack of standardized protocols for exosome isolation and characterization, leads to inconsistent results and hampers the reproducibility of exosome-based diagnostic tests 51. Finally, while exosome-based diagnostics are non-invasive, their utility is still constrained by the absence of large-scale studies that can validate their diagnostic accuracy and sensitivity across diverse patient populations. These challenges highlight the need for further research into standardized methodologies, improved detection sensitivity, and extensive clinical validation to fully harness the potential of exosome-based diagnostics in cancer detection 52.

## 4. Exosomal biomarkers in tumor diagnostics

**Figure 4:**
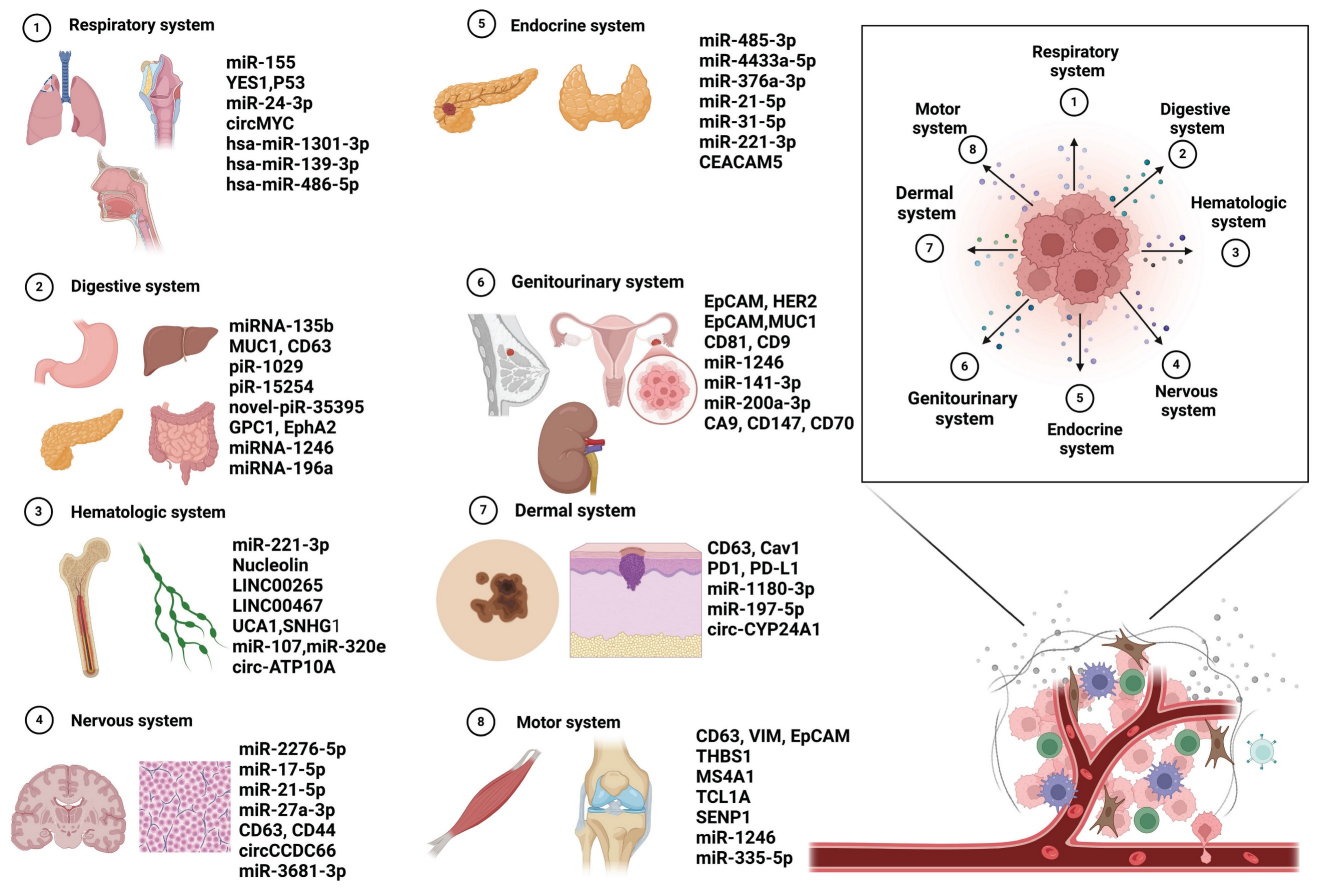
Exosomal biomarkers of systemic tumors. Application in tumors of different systems and some examples of biomarkers. Created with BioRender.com.

Cancer continues to be a major global health issue, where early detection is key to enhancing patient survival and optimizing treatment outcomes. Fueled by rapid advancements in molecular biology and nanotechnology, the scientific community has progressively unraveled the complexities of intercellular communication networks. In this landscape, exosomes--nanoscale vesicles secreted by cells--have emerged as powerful diagnostic tools due to their rich content of nucleic acids, proteins, and other biomolecules. Exosomal biomarkers offer exceptional sensitivity and specificity, and their utilization in non-invasive liquid biopsy techniques enables dynamic monitoring. This breakthrough paves the way for a new era in the early detection of cancer, promising more accurate and timely diagnoses 53 (Figure 4).

### 4.1. Respiratory system

**Lung cancer** is the most common and deadly respiratory malignancy, accounting for the highest incidence and mortality rates globally. It is mainly divided into non-small cell lung cancer (NSCLC) and small cell lung cancer (SCLC), with NSCLC making up around 85% of cases 54. Lung cancer's high prevalence and poor prognosis make it a major challenge in respiratory oncology. Its development involves a complex process driven by genetic and environmental factors, as well as changes in molecular pathways 55. Advances in molecular biology have helped identify genetic mutations and disrupted signaling pathways, enabling targeted therapies and precision medicine. Despite these advancements, survival rates for advanced lung cancer remain low 56. Current diagnostic limitations include the absence of early symptoms, leading to diagnoses at advanced stages, often missing the optimal treatment window. Imaging tools like chest X-rays and CT scans have limited sensitivity and specificity, leading to false results and affecting diagnostic accuracy. Diagnosis often relies on invasive tissue biopsies, which can be complex and risky, discouraging some patients. While biomarker detection holds promise for classification and treatment guidance, their clinical utility is still limited 57. Furthermore, the high cost and limited access to molecular diagnostics and personalized therapies restrict their widespread use. Uneven distribution of medical resources can also delay diagnoses, further impacting prognosis. Thus, improving early screening, non-invasive diagnostic methods, biomarker development, and resource allocation is essential to enhance diagnosis and treatment outcomes 58. Exosomes, as biomarkers, provide a more convenient and effective option for the early diagnosis of lung cancer.

Yunpeng Fan *et al.* developed the Integrated Concentration and Determination System of Exosomes (ICDSE), which combines engineered red blood cells (RBCs) and a plasmonic sensor for efficient screening of exosomal miR-155 in NSCLC patients. The RBCs, functionalized with CD63-specific aptamers, capture exosomes, which are then isolated using simple centrifugation. A plasmonic sensor amplifies the miRNA signal with a detection limit of 2.03 fM, offering a fast, cost-effective, and highly sensitive approach for exosome-based liquid biopsies and early cancer diagnosis 59. Esther Redin *et al.* identified YES1 as a novel oncogenic target and biomarker in SCLC. YES1, detectable in exosomes, correlates with tumor characteristics and prognosis, with high expression linked to shorter progression-free survival (PFS) and overall survival (OS). Its role in predicting sensitivity to therapies like CH6953755 and dasatinib enhances its clinical utility, offering a real-time disease monitoring tool and personalized treatment strategy 60. Li Ming's team showed that seven autoantibodies detected on plasma-derived small extracellular vesicles (sEVs) outperform serum autoantibodies in early lung cancer diagnosis, with improved sensitivity and specificity. These sEV-associated autoantibodies are enriched by tumor-associated antigens (TAAs) and may aid immune evasion in lung cancer. This highlights the potential of sEVs as non-invasive biomarkers for early lung cancer screening and their role in immune modulation 61. Another study combined Exosome-SERS with Artificial Intelligence (AI) to detect early-stage cancers, including lung, breast, and colon cancers, with high sensitivity (90.2%) and specificity (94.4%). The system also identified the tissue of origin with an AUC of 0.945, offering a rapid, label-free, and cost-effective diagnostic tool for clinical use, supporting precision medicine in exosome-based cancer research 62.

**Nasopharyngeal carcinoma (NPC)** is a malignancy of the nasopharynx, often linked to Epstein-Barr virus (EBV) infection. It is most common in Southeast Asia, southern China, and North Africa, with risk factors such as genetic predisposition, diet, and environmental exposures. Most cases are undifferentiated carcinoma, with symptoms like nasal obstruction, nosebleeds, hearing loss, and enlarged cervical lymph nodes. Diagnosis involves endoscopy, imaging, biopsy, and plasma EBV DNA as a biomarker. Treatment typically involves radiation and chemotherapy, with advancements like IMRT improving outcomes 63. Despite advances in diagnostic methods, early detection of NPC remains challenging. The nasopharynx's anatomical location complicates detection during routine exams, often leading to delayed diagnosis until symptoms become apparent. Initial symptoms such as nasal congestion or mild hearing loss are commonly mistaken for conditions like sinusitis. Although MRI and CT scans are sensitive, they may not reliably differentiate early-stage NPC from benign lesions 64. Biopsy, the gold standard, is invasive and may not be performed quickly, particularly in asymptomatic cases. Biomarkers like EBV DNA improve diagnostic accuracy but remain limited by availability and cost. These challenges underscore the need for better screening, more specific biomarkers, and greater public awareness for earlier detection and improved patient outcomes 65. Exosomal biomarkers offer a promising new avenue for the early diagnosis of NPC.

Chuanben Chen *et al.* identify potential plasma biomarkers for early-stage NPC by analyzing exosomal miRNAs via RNA sequencing, revealing 31 differentially expressed miRNAs (21 upregulated, 10 downregulated). Notably, hsa-miR-1301-3p is significantly upregulated and validated as a promising diagnostic biomarker. Bioinformatics analysis indicates that the target genes of these miRNAs are enriched in cancer-related pathways, such as PI3K-Akt and MAPK signaling. These findings suggest that exosomal miRNAs, particularly hsa-miR-1301-3p, could serve as non-invasive biomarkers for early NPC diagnosis 66. Jiang Li *et al.* show that exosomal miR-24-3p, enriched in NPC-derived exosomes, suppresses T-cell proliferation and differentiation by targeting FGF11 and modulating ERK and STAT pathways. Hypoxia enhances miR-24-3p expression, amplifying its immunosuppressive effects.

Elevated serum miR-24-3p levels correlate with worse disease-free survival, while higher FGF11 expression associates with improved prognosis and greater densities of tumor-infiltrating lymphocytes. These findings suggest miR-24-3p and FGF11 as potential prognostic biomarkers and therapeutic targets for NPC 67. Rong Gui *et al.* reveal exosomal circMYC as a potential biomarker and therapeutic target for radioresistant NPC. CircMYC is upregulated in the serum exosomes of NPC patients, especially those with radioresistance, and correlates with larger tumor size, lymph node metastasis, advanced TNM stage, poor survival outcomes, and higher recurrence rates. Functional studies show that circMYC promotes proliferation and reduces radiosensitivity by sponging tumor-suppressive miRNAs, influencing AGO1 and CRY2 pathways. ROC analysis confirms circMYC's high sensitivity and specificity in distinguishing radioresistant from radiosensitive NPC patients, highlighting its clinical potential 68.

Exosomal biomarkers are revolutionizing the early diagnosis, prognosis, and therapeutic monitoring of respiratory tumors, including lung cancer, NPC, and LSCC (Table 2). These vesicles, enriched with miRNAs (e.g., miR-21), lncRNAs (e.g., HOTAIR), circRNAs (e.g., circMYC), and proteins (e.g., IGFBP7), offer high stability and specificity for liquid biopsies. With diagnostic accuracies often exceeding AUC 0.95, they provide insights into tumor aggressiveness, metastasis, and survival. Functionally, they affect key pathways like PI3K-Akt and MAPK, driving tumor progression and therapy resistance. Emerging technologies like Exosome-SERS-AI enable multi-cancer detection. Despite challenges in standardizing methods, exosomal biomarkers hold great promise for improving early detection, risk stratification, and personalized treatment in respiratory tumors.

### 4.2. Digestive system

**Gastric cancer (GC)** ranks as the fifth most prevalent type of cancer and is responsible for the third highest number of cancer-related deaths worldwide. Over 80% of GC patients are diagnosed at a late stage 69. The late diagnosis of GC significantly contributes to its high mortality rate. Currently, GC detection relies on endoscopy and histological biopsy, which are invasive, time-consuming, and expensive. The delay in diagnosis and limited screening among high-risk populations have drawn considerable attention. Consequently, developing rapid and early diagnostic methods is crucial for improving GC patient survival 70. In recent times, liquid biopsy using specific gastric biomarkers has gained recognition as a less invasive alternative to traditional biopsies. Body fluids offer a range of potential biomarkers, including proteins, DNAs, RNAs, and extracellular vesicles, for GC detection. The characteristics of exosomes render them a highly potential option for liquid biopsy applications 71.

Qiang Ma *et al.* developed a novel electrochemiluminescence (ECL) sensor using MoS2 QDs-MXene heterostructures and Au NPs@biomimetic lipid layers to detect miRNA-135b, a gastric cancer biomarker in exosomes. The MoS2 QDs-MXene heterostructure amplifies the signal, while the Au NPs@biomimetic lipid layer provides a specific, antifouling platform for miRNA capture. The sensor achieves highly sensitive detection with a limit of 10 fM, offering a rapid, non-invasive method for gastric cancer diagnosis and monitoring 72. Additionally, Qiang Ma *et al.* developed a magnetoplasmonic metasurface-modulated ECL sensor for ultrasensitive detection of gastric cancer-derived extracellular vesicles (EVs). Using Fe3O4@Au yolk-shell nanoparticles and MUC1 aptamers for recognition, this system enhances ECL signals and achieves high sensitivity (detection limit of 200 particles/mL). This approach provides a powerful tool for early gastric cancer diagnosis and monitoring, particularly for peritoneal metastasis 73. Z. Li *et al.* introduced a droplet digital branched rolling circle amplification (ddBRCA) biosensor for ultrasensitive detection of gastric cancer-derived EVs. The platform uses stem-loop hairpin DNA (APP) with MUC1-specific aptamers to recognize target EVs, triggering a BRCA reaction that amplifies fluorescence signals. This method allows single-EV analysis with a detection limit of 12 particles/mL and provides a rapid, highly sensitive, and clinically validated tool for non-invasive gastric cancer diagnostics 74.

**Hepatocellular carcinoma (HCC),** which accounts for 75% to 85% of primary liver cancer cases, is the most common form of liver cancer and ranks as the third leading cause of cancer-related mortality globally. Diagnosis typically involves imaging and histopathological biopsies. However, up to 30% of HCC patients show negative alpha-fetoprotein (AFP) results, limiting AFP's sensitivity and specificity for screening 75. Imaging has high specificity but relatively low sensitivity, making detection of small tumors difficult. Histopathological biopsies, although informative, are invasive and prone to false negatives. As early symptoms and specific biomarkers are often absent, most HCC patients are diagnosed at advanced stages, limiting treatment efficacy. Early detection is crucial for improving patient outcomes 76. The occurrence and progression of liver cancer is complex, and its molecular mechanisms remain unclear. While viral infections, alcohol, and non-alcoholic hepatotoxicity are major contributors to HCC, the full pathogenesis is not completely understood. Recent *in vivo* and *in vitro* studies suggest that exosomes play a critical role in the initiation, progression, diagnosis, and treatment of HCC, positioning them as promising candidates for novel biomarkers 77.

Xiaobing Zhang *et al.* discovered that serum exosome-derived PIWI-interacting RNAs (piRNAs) are promising biomarkers for HCC diagnosis. Among 253 differentially expressed piRNAs, five (piR-1029, piR-15254, novel-piR-35395, novel-piR-43597, and novel-piR-32132) were highly upregulated in HCC patients, showing superior diagnostic accuracy (AUROC up to 0.986) compared to AFP, even in low tumor burden cases. Unique base distribution patterns in HCC-derived piRNAs may help differentiate HCC from non-tumor donors. This highlights piRNAs' potential as stable and specific biomarkers for non-invasive early HCC detection 78. Minqiang Lu *et al.* identified the exosomal lncRNA THEMIS2-211 as a tumor-derived biomarker significantly upregulated in HCC, with diagnostic and prognostic value. THEMIS2-211 acts as a competing endogenous RNA (ceRNA), sponging miR-940 to upregulate SPOCK1, driving tumor proliferation, invasion, and EMT. Its exosomal origin ensures stability, outperforming AFP in early-stage HCC diagnosis. Targeting the THEMIS2-211/miR-940/SPOCK1 axis could offer new therapeutic strategies for HCC 79. Fubing Wang *et al.* developed a SiO₂-coated 3D hierarchical porous chip (SiO₂-chip) for efficient exosome enrichment, identifying lncRNAs LUCAT-1 and EGFR-AS1 as specific biomarkers for early HCC detection and monitoring. The SiO₂-chip captures exosomes using a 3D porous scaffold, enabling rapid isolation from minimal plasma samples. These lncRNAs show superior diagnostic accuracy (AUC: 0.947) and prognostic value, correlating with tumor progression and poor survival. The platform offers high sensitivity (detection limit: 10,000 particles/mL), speed (under 10 minutes), and robustness, making it a powerful tool for non-invasive HCC diagnostics and treatment monitoring 80.

**Pancreatic cancer,** particularly pancreatic ductal adenocarcinoma (PDAC), is a highly aggressive malignancy with one of the poorest prognoses. It is often asymptomatic in its early stages, leading to late-stage diagnosis when the disease is typically advanced or metastatic. Major risk factors include obesity, smoking, chronic pancreatitis, diabetes, and a family history of the disease 81. Despite advances in surgery, chemotherapy, and targeted therapies, the five-year survival rate remains low due to late detection. Diagnosis of pancreatic cancer is challenging due to the asymptomatic nature of early stages and the lack of specific biomarkers. Current diagnostic methods, such as imaging (CT, MRI, and endoscopic ultrasound) and serum biomarkers like CA19-9, have limitations. Imaging may fail to detect small or early-stage tumors, and CA19-9 lacks sensitivity and specificity, as its levels can be elevated in benign conditions like pancreatitis. Invasive biopsies are not always feasible due to the tumor's location 82. These limitations lead to delayed diagnosis, emphasizing the need for more accurate, non-invasive diagnostic tools for early detection and improved outcomes in this challenging cancer.

Jianlin Shi *et al.* developed a nanoliquid biopsy (nLB) assay using dual biomarkers, GPC1 and EphA2, to detect pancreatic cancer tumor exosomes (T-Exos). With magnetic nanoparticles for isolation and gold nanoparticles for signal amplification, the assay achieves high sensitivity (78 pg/mL detection limit) and 100% specificity. It provides precise early-stage diagnosis (AUC: 1.0) and reliable tumor monitoring, making it a promising non-invasive tool for clinical use 83. Lingling Wu *et al.* created a multiplex detection strategy for tumor-derived extracellular vesicle microRNAs (tEV-miRNAs) using encoded-targeted fusion beads (ETFBs) and aptamer-modified supported lipid bilayers (SLBs). This system captures and profiles six key miRNAs from plasma without exosome isolation, achieving 98% diagnostic accuracy and rapid processing (within 2 hours), making it a non-invasive and efficient tool for early pancreatic cancer detection 84. L. James Lee *et al.* introduced an Immune Lipoplex Nanoparticle (ILN) biochip for detecting GPC1 mRNA in exosomes and GPC1 protein in tumor-associated microvesicles (tMVs). This dual biomarker approach achieves high diagnostic accuracy (AUC: 0.960) and provides prognostic value, correlating low GPC1 levels with prolonged survival in late-stage PDAC patients. It offers high sensitivity and rapid processing, making it a powerful tool for PDAC diagnosis and prognosis 85.

**Colorectal cancer (CRC)** is a common and life-threatening malignancy that typically starts as benign polyps, which can become cancerous over time if not removed. Risk factors include age, high consumption of processed or red meats, low fiber intake, obesity, smoking, excessive alcohol, inflammatory bowel disease, and a family history of CRC 86. It is worth noting that although older patients with pT4 disease are more prone to severe postoperative complications, there is no consensus on whether age affects survival outcomes. The prognosis of older patients may be confounded by differences in stage at presentation, tumor location, preexisting comorbidities, and the type of treatment received 87. Symptoms include changes in bowel habits, abdominal pain, blood in the stool, fatigue, and weight loss. Regular screening, such as colonoscopy and stool-based tests, is essential for early detection and prevention. Current diagnostic methods for CRC have limitations. Colonoscopy is the gold standard but is invasive, time-consuming, and requires bowel preparation, deterring some patients 88. Non-invasive stool-based tests, such as fecal immunochemical tests (FIT) and stool DNA tests, have limited sensitivity and may miss early-stage lesions. Imaging techniques like CT colonography are less invasive but have reduced accuracy for detecting small or flat polyps, often requiring follow-up procedures. Additionally, the lack of early symptoms often leads to delayed diagnoses and poorer outcomes. These limitations highlight the need for more accurate, non-invasive, and patient-friendly diagnostic tools for early CRC detection 89. Exosomes, secreted by cancer cells, play a crucial role in early CRC diagnosis as carriers of tumor-specific biomarkers. These nanosized vesicles can be detected in accessible body fluids like blood and stool, enabling non-invasive liquid biopsy approaches. Exosome-based diagnostics offer high sensitivity and specificity, allowing for the detection of CRC at early stages, even before clinical symptoms arise. This makes exosomes a promising tool for early detection, treatment guidance, and improved patient outcome 90.

Meilin Wang *et al.* identify exosomal circLPAR1 as a stable and specific biomarker for CRC diagnosis and progression. circLPAR1 is significantly downregulated in CRC, with plasma exosomal levels showing an AUC of 0.875, achieving 87.3% sensitivity and 76.3% specificity when combined with traditional biomarkers like CEA and CA19-9. Functionally, circLPAR1 acts as a tumor suppressor by disrupting the METTL3-eIF3h interaction, thereby inhibiting BRD4 translation, a key oncogene in CRC. Its levels correlate with disease progression and decrease after surgical resection, offering both diagnostic and prognostic value. Advantages include its high stability in exosomes, non-invasive detectability in plasma, and its ability to distinguish CRC from other cancers, making it a powerful tool for early detection and treatment monitoring 91. Chu Wang *et al.* developed a rapid and cost-effective EV-based proteomics strategy for early CRC diagnosis. By using DSPE-functionalized beads, EVs were efficiently isolated from plasma within 10 minutes, followed by proteomic analysis using SP3 technology and DIA-MS. A machine learning model identified 10 key EV protein biomarkers, achieving 89.3% diagnostic accuracy and an AUC of over 0.94. The mechanism leverages hydrophobic interactions for specific EV capture and integrates mass spectrometry data with machine learning for precise biomarker identification. This approach is non-invasive, scalable, and highly accurate, offering significant potential for early CRC detection and broader clinical applications 92. Shutian Zhang *et al.* identified a 10-gene sEV-RNA signature for the early detection of T1a stage CRC and advanced adenoma (AA) through whole-transcriptomic profiling of circulating sEV-derived RNAs. This signature, enriched in cancer-related pathways, achieved high diagnostic accuracy (AUC: 0.94, 99.0% sensitivity, 79.3% specificity) and was validated in independent cohorts. The mechanism leverages sEVs' ability to protect RNAs from degradation, enabling robust liquid biopsy-based detection. Its advantages include early detection of precancerous and early-stage CRC lesions, non-invasiveness, high stability of sEV-RNAs, and scalability for large-scale clinical applications, addressing critical gaps in CRC screening 93.

Exosomal biomarkers are transforming the non-invasive diagnosis of digestive system tumors (Table 3). These biomarkers, such as serum exosome-derived piRNAs and dual markers like GPC1 and EphA2, enable early detection with high sensitivity and specificity, outperforming traditional methods like CA19-9 and AFP. Their integration into liquid biopsy platforms offers a patient-friendly and minimally invasive alternative to conventional diagnostics, allowing for routine screening, instantaneous tracking of treatment response, and early detection of recurrence. With their stability, scalability, and ability to provide biological insights, exosomal biomarkers are poised to revolutionize cancer diagnosis and management, improving outcomes for patients with digestive system tumors.

### 4.3. Hematologic system

**Leukemia** is a type of hematologic malignancies marked by the uncontrolled growth of abnormal white blood cells in the bone marrow and blood. It disrupts the normal production of blood cells, leading to symptoms such as anemia, infections, bleeding tendencies, fatigue, and bone pain. Leukemia is broadly classified into acute and chronic forms, with further subtypes such as acute lymphoblastic leukemia (ALL), acute myeloid leukemia (AML), chronic lymphocytic leukemia (CLL), and chronic myeloid leukemia (CML) 94. Risk factors include genetic predispositions, exposure to radiation or certain chemicals, and prior chemotherapy. Diagnosis involves blood tests, bone marrow examination, and molecular studies. Advances in targeted therapies, immunotherapies, and stem cell transplantation have significantly improved outcomes, especially when leukemia is detected early and treated promptly. The diagnosis of leukemia faces several limitations despite advances in diagnostic tools. While blood tests and bone marrow examinations are essential for identifying abnormal cells, these methods are invasive and can be uncomfortable for patients 95. Molecular and cytogenetic analyses provide critical insights but are time-consuming and require specialized equipment, limiting their accessibility in resource-constrained settings. Additionally, early-stage or indolent forms of leukemia, such as CLL, may be asymptomatic and go undetected during routine screenings. The lack of specific biomarkers for certain subtypes further complicates early diagnosis and differentiation between similar hematologic disorders. These challenges underscore the need for more precise, non-invasive, and rapid diagnostic approaches to improve leukemia detection and patient outcomes 96.

Hui Cheng *et al.* discovered that sEVs derived from AML cells promote leukemogenesis by transferring miR-221-3p, a key microRNA. miR-221-3p enhances AML cell proliferation and survival by targeting Gbp2, thereby activating the PI3K/AKT signaling pathway, while also impairing the function and differentiation of hematopoietic stem and progenitor cells (HSPCs). The findings highlight the role of AML-sEVs in creating a leukemia-permissive bone marrow niche. This mechanism not only offers insights into AML progression but also positions miR-221-3p as a potential biomarker for diagnosis and a therapeutic target to disrupt the leukemogenic microenvironment, providing a novel avenue for AML management 97. Lin Huang *et al.* developed a highly sensitive colorimetric biosensor for detecting leukemia-derived exosomes by targeting nucleolin, a protein enriched on exosome surfaces. The mechanism combines rolling circle amplification (RCA) with dual signal amplification using gold nanoparticles and horseradish peroxidase (GNPs-HRP) to produce a visible colorimetric response. This biosensor achieves a detection limit as low as 100 particles/μL and requires minimal sample volume (40 μL). Its advantages include high specificity, non-invasiveness, cost-effectiveness, and simplicity, as it allows visual detection without complex instruments. These features make it a promising tool for early leukemia diagnosis and treatment monitoring 98. Ling Zhang *et al.* identified four plasma exosomal lncRNAs--UCA1, LINC00467, LINC00265 and SNHG1--as potential biomarkers for AML diagnosis and treatment monitoring. These lncRNAs, with distinct expression patterns in AML patients, allow differentiation from healthy controls and tracking of treatment response, particularly during chemotherapy or stem cell transplantation. The mechanism involves the stable encapsulation of lncRNAs within exosomes, protecting them from degradation and enabling reliable detection. Advantages include non-invasive sampling, high stability, real-time monitoring of disease progression, and improved diagnostic accuracy when combining multiple lncRNAs, making them a promising tool for AML management 99.

**Multiple myeloma** (MM) is a malignancy of plasma cell disorder characterized by the clonal proliferation of abnormal plasma cells in the bone marrow. These cells produce excessive monoclonal immunoglobulins (M-proteins), leading to various complications such as bone lesions, anemia, renal dysfunction, hypercalcemia, and an increased risk of infections. MM typically develops in older adults and is often preceded by precursor conditions like monoclonal gammopathy of undetermined significance (MGUS) or smoldering myeloma. Diagnosis involves blood and urine tests (for M-proteins), bone marrow biopsy, and imaging studies to detect bone damage 100. Advances in treatments, including proteasome inhibitors, immunomodulatory drugs, monoclonal antibodies, and autologous stem cell transplantation, have significantly improved outcomes, but MM remains incurable, with relapses common. Early detection and tailored therapeutic strategies are essential for managing the disease and enhancing patient survival and quality of life.

Chen Kuisheng *et al.* discovered that cancer-associated fibroblast (CAF)-derived exosomal miR-21 plays a critical role in promoting angiogenesis in MM by enhancing endothelial cell proliferation, migration, and tube formation. The mechanism involves miR-21 activating pro-angiogenic pathways in MM endothelial cells and facilitating the transformation of normal fibroblasts into CAFs, which further secrete pro-angiogenic exosomes. This exosomal miR-21 is stable, specific to the tumor microenvironment, and detectable in circulation, making it a promising biomarker for MM progression and a therapeutic target to suppress tumor angiogenesis, offering non-invasive diagnostic and treatment potential 101. Xing Cui *et al.* identify circ-ATP10A, an exosome-enriched circular RNA, as a novel biomarker for MM. circ-ATP10A promotes angiogenesis by acting as a miRNA sponge, sequestering miRNAs like hsa-miR-6758-3p and modulating the expression of angiogenesis-related genes such as VEGFB and HIF1A. It is significantly upregulated in MM patients and correlates with increased bone marrow microvessel density and poor survival outcomes. Advantages of circ-ATP10A include its high diagnostic accuracy (AUC: 0.854), non-invasive detection via serum exosomes, stability in circulation, and prognostic value, making it a promising tool for MM diagnosis, prognosis, and as a therapeutic target to inhibit angiogenesis-driven tumor progression 102.Yanwei Luo *et al.* identified LRG1, a protein enriched in platelet-derived exosomes, as a key promoter of MM progression. LRG1 interacts with OLFM4 to activate EMT and angiogenesis, enhancing MM cell proliferation, tumor invasiveness, and vascular formation. Elevated levels of exosomal LRG1 in MM patients correlate with advanced disease stages and poor survival outcomes, highlighting its role as a biomarker and therapeutic target. Advantages of exosomal LRG1 include its high specificity to MM, non-invasive detection through liquid biopsy, and potential for targeted therapies to disrupt its tumor-promoting effects, offering new strategies for MM diagnosis and treatment 103.

**Lymphoma** is a form of cancer that originates in the lymphatic system, which plays a crucial role in immune defense. It occurs when lymphocytes, a type of white blood cell, grow uncontrollably and form tumors in lymph nodes, spleen, bone marrow, or other organs. Lymphoma is broadly classified into Hodgkin lymphoma (HL) and non-Hodgkin lymphoma (NHL), with NHL being more common and diverse. Risk factors include weakened immunity, certain infections (e.g., Epstein-Barr virus or HIV), and exposure to radiation or chemicals. Symptoms often include fatigue, fever, painless swelling of lymph nodes, night sweats, and unexplained weight loss 104. Diagnosis typically involves imaging, blood tests, and lymph node biopsy. Advances in targeted therapies, immunotherapy, and stem cell transplantation have significantly improved outcomes, with many forms of lymphoma being treatable or even curable, especially when detected early. The diagnosis of lymphoma faces several limitations despite advances in medical technology. Traditional methods like lymph node biopsy, while accurate, are invasive and can be uncomfortable for patients. Imaging techniques such as CT, MRI, or PET scans are useful but may fail to detect very small lesions or distinguish lymphoma from benign conditions 105. Blood tests, while supportive, lack the sensitivity and specificity to definitively diagnose lymphoma. Furthermore, the diverse subtypes of lymphoma, particularly in NHL, complicate diagnosis and require advanced immunophenotyping or molecular testing, which may not be readily available in all clinical settings. These challenges highlight the need for non-invasive, specific, and accessible diagnostic tools to improve early detection and accurate classification of lymphoma subtypes 106.

Seok Jin Kim *et al.* identify serum-derived exosomal miR-320e, miR-21-5p and miR-4454 as key prognostic biomarkers for extranodal natural killer/T-cell lymphoma (ENKTL). These miRNAs are upregulated in advanced disease stages, associated with poor survival, and linked to treatment resistance, particularly to etoposide. Mechanistically, they promote tumor progression by enhancing inflammatory cytokine secretion, inducing M2-like macrophage polarization, and creating an immune-suppressive tumor microenvironment. Advantages include their stability within exosomes, non-invasive detectability via liquid biopsy, strong correlation with prognosis and treatment response, and potential as therapeutic targets to overcome resistance and improve patient outcomes 107. Xin Wang *et al.* identified exosomal miR-107 as a tumor suppressor in diffuse large B-cell lymphoma (DLBCL), with significantly reduced levels in patients correlating with advanced disease and poor survival. Mechanistically, miR-107 targets and downregulates the oncogenic protein 14-3-3η, disrupting PI3K/Akt signaling and cell cycle regulation, thereby inhibiting tumor cell proliferation, invasion, and promoting apoptosis. Advantages of miR-107 include its high diagnostic accuracy (AUC: 0.854), non-invasive detectability via plasma exosomes, stability in circulation, prognostic relevance, and therapeutic potential as a target to suppress tumor progression and overcome resistance in DLBCL 108.

The findings highlight the transformative potential of exosomal biomarkers in diagnosing and managing hematologic malignancies, including AML, MM, ENKTL, and DLBCL (Table 4). These studies demonstrate the utility of exosomal microRNAs (e.g., miR-221-3p, miR-107) and long non-coding RNAs (e.g., LINC00265) for early detection, prognostic stratification, and treatment monitoring with high sensitivity and specificity. Exosome-based liquid biopsies provide a non-invasive, stable, and reliable alternative to traditional diagnostic methods like bone marrow biopsies. Additionally, these biomarkers reveal tumor-promoting mechanisms, such as angiogenesis and immune modulation, offering therapeutic insights. Collectively, these advancements pave the way for precision medicine and improved outcomes in hematologic oncology.

### 4.4. Nervous system

Gliomas are the most common primary brain tumors, originating from neural stem or progenitor cells that acquire tumor-initiating genetic mutations. These tumors are classified under the World Health Organization (WHO) grading system, ranging from Grade 1 (benign) to Grade 4 (highly malignant). Adult diffuse gliomas, such as isocitrate dehydrogenase (IDH)-mutant astrocytomas and oligodendrogliomas, often have better prognoses, while IDH-wild-type glioblastomas are highly aggressive and carry poor survival outcomes. Pediatric gliomas include both low-grade and high-grade forms, with distinct molecular alterations, such as histone mutations in midline gliomas, leading to their aggressive behavior 109. The development of gliomas involves genetic and epigenetic changes, including mutations in IDH, TP53, and ATRX, alongside alterations in pathways like PI3K/AKT and MAPK, contributing to tumor growth and resistance to therapy. Gliomas are challenging to treat due to their invasive nature, resistance mechanisms, and immunosuppressive microenvironment. Standard therapies include surgery, radiotherapy, and chemotherapy, but emerging molecularly targeted therapies offer hope for improved outcomes 110. Advancements in molecular profiling and biomarker discovery have enhanced diagnostic accuracy, enabling personalized treatment approaches. In particular, the total number of lncRNAs has been steadily increasing, thanks to more sensitive detection methods. Today, the number of lncRNAs surpasses that of all protein-coding genes. Primarily transcribed by RNA polymerase II, lncRNAs undergo various post-transcriptional modifications. They are found in multiple cellular compartments, including the nucleus, nucleolus, cytoplasm, and mitochondria. Growing evidence suggests a mechanistic link between lncRNA dysregulation and numerous human diseases, including cancer, positioning lncRNAs as promising therapeutic targets and biomarkers 111. However, challenges in early detection, the complexity of molecular subtypes, and limited treatment efficacy for high-grade gliomas underscore the need for continued research and innovation in glioma management 112. The emergence of exosomes as biomarkers has provided new possibilities for the early detection of glioma.

Shiguang Zhao *et al.* identified exosomal miR-2276-5p as a promising glioma biomarker, with low expression in glioma patients, particularly high-grade ones, and poor survival correlation. It targets RAB13, impacting tumor progression and the JAK/STAT3 pathway, and shows strong diagnostic accuracy (AUC: 0.8107). miR-2276-5p's non-invasive detection via plasma exosomes highlights its potential as both a biomarker and therapeutic target 113. Huilin Shao *et al.* introduced EZ-READ, a blood-based glioblastoma (GB) diagnostic platform, detecting mRNA and miRNA signatures from EVs with high sensitivity (detection limit: 9 RNA copies) and accuracy (AUC: 0.897). EZ-READ offers a rapid, non-invasive alternative to PCR, ideal for real-time monitoring and personalized medicine 114. Yanlin Song *et al.* developed a multiplex optical biochip for exosome detection in glioblastoma, using nanochains to capture exosomes and amplify signals. It can simultaneously detect multiple biomarkers (e.g., CD44, CD133) within 30 minutes, providing a cost-effective, non-invasive approach for early diagnosis and personalized treatment 115. Guan Sun *et al.* identified circBTG2, enriched in exosomes from RBP-J-overexpressed macrophages, as a glioma biomarker. It regulates key oncogenic pathways through the circBTG2/miR-25-3p/PTEN axis, offering both diagnostic and therapeutic potential for liquid biopsy applications and personalized treatment 116.

**Pituitary adenomas (PAs)** are typically benign tumors of the pituitary gland, classified by size into microadenomas (<10 mm) and macroadenomas (>10 mm), and by hormone activity into functional (hormone-secreting) and non-functional (non-secreting) types. Functional adenomas cause hormone-related syndromes, such as prolactinomas (hyperprolactinemia), acromegaly (excess growth hormone), or Cushing's disease (excess cortisol), while non-functional pituitary adenomas (NFPAs) often present with symptoms like headaches or visual disturbances due to compression of surrounding structures 117. Diagnosis involves hormonal tests, MRI imaging, and sometimes visual field assessments. Treatment options include medical therapy (e.g., dopamine agonists for prolactinomas), surgery (typically via a transsphenoidal approach), and, in some cases, radiation therapy. While benign, pituitary adenomas can cause significant health issues, underscoring the importance of early diagnosis and treatment. The diagnosis of PAs faces challenges due to their diverse presentations and subtle early symptoms. Functional adenomas may cause hormonal syndromes like Cushing's disease or acromegaly, but these symptoms often develop gradually and overlap with other conditions, delaying recognition. NFPAs are typically asymptomatic until they grow large enough to cause headaches or visual disturbances. Small microadenomas can be difficult to detect on imaging, and hormonal tests may yield false positives or negatives due to confounding factors. Differentiating benign adenomas from malignant tumors is also challenging without invasive procedures. These issues emphasize the need for better imaging techniques, specific biomarkers, and improved clinical awareness 118. Exosomes, as biomarkers, provide a convenient and effective approach for the diagnosis of PAs.

Peizhi Zhou *et al.* highlight the potential of exosomal miRNA profiling for non-invasive diagnosis and prognosis of non-functioning pituitary adenomas (NFPAs). hsa-miR-486-5p was identified as a promising biomarker with high diagnostic accuracy and strong predictive value for tumor recurrence or progression. Next-generation sequencing revealed 54 differentially expressed miRNAs, with bioinformatics suggesting that hsa-miR-486-5p regulates tumor progression via the MAPK signaling pathway, DNA repair, and epigenetic mechanisms. Higher levels of hsa-miR-486-5p were associated with worse progression-free survival, supporting its use for personalized treatment strategies. Further validation in larger cohorts is needed 119. Weiping Liu *et al.* identified serum exosomal circCCDC66 as a promising biomarker for the diagnosis and prognosis of pituitary adenomas (PAs). circCCDC66 was significantly upregulated in PA patients, showing strong correlation between exosomal and tissue levels. It demonstrated high diagnostic accuracy (AUC: 0.8719) with 80% sensitivity and 84% specificity. Post-surgical levels decreased but increased at recurrence, aiding in tumor monitoring. Lower circCCDC66 expression correlated with longer progression-free survival, suggesting its role in early detection and recurrence prediction. Further large-scale validation studies are needed 120. Guojun Zhang *et al.* emphasized the potential of EMT-related markers in serum exosomes as non-invasive biomarkers for diagnosing and monitoring invasive pituitary adenomas (IPAs). Invasive PAs showed higher mesenchymal marker expression (N-cadherin, vimentin) and lower epithelial marker expression (E-cadherin, Epcam) in serum exosomes and tumor tissues. The N-cadherin/Epcam ratio was significantly elevated in invasive cases, aiding differentiation from non-invasive adenomas. The TGF-β/Smad signaling pathway was also found to negatively regulate EMT in pituitary adenomas, with therapeutic implications. These findings suggest that EMT-related exosomal markers could be valuable for non-invasive diagnosis and monitoring of IPA progression, though larger studies are needed for validation 121.

The studies highlight the potential of exosomal biomarkers for diagnosing, prognosing, and monitoring nervous system tumors, including gliomas, glioblastomas, and PCNSL (Table 5). Exosomal miRNAs like miR-2276-5p and circBTG2 are promising for glioma diagnosis and progression, while platforms like EZ-READ and biochips enable non-invasive glioblastoma subtyping and monitoring. These biomarkers allow non-invasive sampling, improved sensitivity, and real-time monitoring, though more clinical validation is needed.

### 4.5. Endocrine system

**Thyroid cancer,** the most common endocrine malignancy, includes four types: papillary thyroid cancer (PTC), follicular thyroid cancer (FTC), medullary thyroid cancer (MTC), and anaplastic thyroid cancer (ATC). Symptoms include a painless neck lump, hoarseness, and difficulty swallowing or breathing. Diagnosis involves imaging, biopsy, and thyroid function tests, with treatment typically including surgery, radioactive iodine, and sometimes chemotherapy for advanced cases 122. PTC and FTC generally have an excellent prognosis with early detection. Despite advances in diagnostic techniques, thyroid cancer diagnosis faces challenges. Early-stage thyroid cancer is often asymptomatic, making detection difficult without routine screening 123. Thyroid ultrasound, while essential for evaluating nodules, often cannot distinguish benign from malignant ones, leading to unnecessary biopsies and increased costs 124. FNAB can yield indeterminate cytology (e.g., Bethesda categories III and IV), creating uncertainty about the next steps 125. Molecular testing improves accuracy but is limited by high costs and inconsistent availability 126. Diagnosing aggressive forms like ATC or small metastases remains difficult, as current imaging may miss microscopic disease. These issues highlight the need for better diagnostic tools, including precise imaging, advanced molecular biomarkers, and risk stratification systems.

Yongsheng Zhao *et al.* identified plasma exosomal miR-485-3p and miR-4433a-5p as non-invasive biomarkers for diagnosing and stratifying papillary thyroid cancer (PTC). MiR-485-3p showed the highest diagnostic accuracy (AUC: 0.8581, sensitivity: 85.42%, specificity: 73.33%) and was linked to high-risk features like larger tumor size, advanced stage, BRAF mutation, and lymph node metastasis, making these miRNAs valuable for distinguishing PTC and identifying aggressive disease 127. Pei-Jie Huang *et al.* identified urinary exosomal TIMP and Angiopoietin-1 as biomarkers for predicting lymph node metastasis (LNM) in well-differentiated thyroid cancer (WDTC). Higher TIMP levels were associated with LNM, and these biomarkers could complement existing tools like MACIS for surgical planning and follow-up 128. Diego Russo *et al.* identified deregulated exosomal miRNAs (miR-31-5p, miR-222-3p, let-7i-3p) as key players in aggressive PTC subtypes. These miRNAs, upregulated in tumor-derived exosomes, are linked to tumorigenic pathways and could serve as biomarkers and therapeutic targets for advanced PTC 129.

**Pancreatic neuroendocrine tumors (pNETs)** are rare neoplasms, making up 1-2% of pancreatic tumors 130. They are classified into functional and non-functional types. Functional pNETs produce hormones, leading to conditions like hypoglycemia in insulinomas, ulcers in gastrinomas, and watery diarrhea in VIPomas. Non-functional pNETs, which are more common, present with abdominal pain, jaundice, or weight loss as the tumor grows or metastasizes 131. Diagnosis involves imaging (CT, MRI, Ga-68 DOTATATE PET/CT) and biomarkers like chromogranin A. Treatment includes surgery for localized tumors, somatostatin analogs for functional tumors, and targeted therapies or peptide receptor radionuclide therapy (PRRT) for advanced cases. Prognosis varies, emphasizing the need for early detection and personalized treatment 132. Diagnosing pNETs is challenging due to their rarity, heterogeneity, and subtle symptoms. Functional pNETs have distinct syndromes but can overlap with common conditions, causing delays in diagnosis. Non-functional pNETs often remain asymptomatic until advanced stages or metastasis, complicating early detection. Imaging techniques, such as CT, MRI, and Ga-68 DOTATATE PET/CT, are essential but may miss small or poorly differentiated tumors. Biochemical markers like chromogranin A lack specificity, with non-tumor factors (e.g., renal impairment, proton pump inhibitors) affecting results 133. Additionally, distinguishing pNETs from other pancreatic cancers and assessing tumor aggressiveness remains difficult. These challenges highlight the need for more sensitive biomarkers, advanced imaging, and integrated diagnostic approaches for better early detection and accurate classification of pNETs.

Qiyun Tang *et al.* reveal that hypoxic pNENts-derived exosomal miR-4488 promotes liver metastasis by reprogramming tumor-associated macrophages (TAMs) into an M2-like phenotype. miR-4488 downregulates RTN3 in macrophages, enhancing fatty acid oxidation (FAO) via FABP5 and activating the PI3K/AKT/mTOR pathway, which drives M2 polarization. These M2 macrophages secrete MMP2, facilitating tumor migration and liver metastasis. *In vivo* models confirmed the metastatic role of hypoxic exosomes, while targeting miR-4488, RTN3, or MMP2 significantly reduced metastasis. These findings suggest that miR-4488 and its downstream targets (RTN3, FAO, MMP2) could serve as promising biomarkers and therapeutic targets to prevent or treat pNEt metastasis 134. The research team also conducted another related study. The study identifies hypoxia-induced exosomal CEACAM5 as a key driver of pancreatic neuroendocrine tumor (pNET) metastasis by promoting tumor-associated macrophage (TAM) M2 polarization and enhancing MMP9 secretion. CEACAM5, upregulated under hypoxic conditions via HIF1α, activates the MAPK/ERK pathway in TAMs, leading to M2 polarization and increased extracellular matrix degradation through MMP9, which facilitates tumor cell migration and invasion. *In vivo* models confirmed that CEACAM5-rich exosomes significantly increased metastatic burden, while knockdown of CEACAM5 or inhibition of exosome secretion and MMP9 reduced metastasis. These findings suggest CEACAM5 as a promising biomarker and therapeutic target for metastatic pNETs, offering new strategies to disrupt tumor progression in this aggressive cancer type 135.

The studies highlight the potential of exosomal biomarkers in diagnosing and managing endocrine tumors (Table 6). Exosomal miRNAs like miR-485-3p and miR-4433a-5p provide strong diagnostic accuracy for PTC, while urinary exosomal proteins (TIMP, Angiopoietin-1) aid in lymph node metastasis assessment for WDTC. In pNETs, exosomal components like miR-4488 and CEACAM5 promote macrophage M2 polarization and metastasis, offering diagnostic and therapeutic potential. These findings emphasize the role of exosomal biomarkers in early detection, risk assessment, and treatment monitoring.

### 4.6. Genitourinary system

**Breast cancer (BC)** is one of the most common malignancies and the leading cause of cancer-related deaths among women worldwide. It arises from the abnormal growth of cells in the breast tissue, often originating in the ducts (ductal carcinoma) or lobules (lobular carcinoma). Key risk factors are genetic predisposition (e.g., BRCA1/2 mutations), hormonal factors, lifestyle influences such as obesity and alcohol consumption, and a family history of the disease 136. Symptoms may include a lump in the breast, changes in breast shape or size, skin dimpling, or nipple discharge. Diagnosis typically involves imaging techniques like mammography and ultrasound, followed by biopsy for histopathological and molecular analysis. Advances in treatment, including surgery, radiotherapy, chemotherapy, targeted therapies, and immunotherapy, have significantly improved survival rates. Early detection through screening programs remains crucial for improving outcomes, as breast cancer is most treatable in its early stages 137. Despite advancements in BC diagnostics, several limitations remain. Imaging techniques like mammography, while effective for early detection, often struggle to distinguish between benign and malignant lesions, leading to false positives and unnecessary biopsies. Additionally, dense breast tissue can obscure tumors, reducing diagnostic accuracy. Biopsy, the gold standard for confirmation, is invasive and may cause patient discomfort. Furthermore, molecular and genetic testing, although valuable for identifying subtypes and guiding treatment, is expensive and not universally accessible. Early-stage cancers or those in non-palpable areas may still go undetected, emphasizing the need for more sensitive, non-invasive, and cost-effective diagnostic tools to address these challenges 138. The emergence of exosomal biomarkers offers promising solutions to these challenges in BC diagnosis.

Jinfeng Zhu *et al.* developed aptamer-based plasmonic metasurfaces (APM) for the label-free detection of breast tumor-derived exosomes, enabling highly sensitive and cost-effective BC diagnosis and molecular subtyping. The mechanism leverages optimized near-field plasmonic effects using thiol-modified aptamers, achieving an improved detection limit of 1.04 × 10⁴ particles/mL and a diagnostic accuracy (AUC: 99.3%) superior to traditional biomarkers like CA153. Advantages include high sensitivity, non-invasive serum-based detection, cost-effectiveness due to aptamer use, simplified workflows without signal amplification, and the ability to classify molecular subtypes such as HER2-positive BC, making it a valuable tool for early detection and personalized treatment strategies 139. Shuqing Sun et al, developed the CRISPR/Cas12a and Aptamer-Chemiluminescence-Based Analysis (CACBA) platform for BC diagnosis by detecting tumor-related proteins (EpCAM and MUC1) on exosome surfaces. The mechanism combines CRISPR/Cas12a for total exosome quantification with aptamer-chemiluminescence assays to calculate the relative abundance of tumor-specific exosomal proteins. This approach achieved exceptional diagnostic accuracy (AUC = 1.00) and sensitivity, with detection limits as low as 1.45 × 10² particles/μl. Advantages include its non-invasive nature, high specificity, robustness against sample variability, cost-effectiveness due to aptamer use, and adaptability for detecting other tumor-related biomarkers, making it a versatile and reliable tool for clinical cancer diagnostics 140. Genxi Li *et al.* developed a biomimetic vesicle system that utilizes cancer cell membrane camouflage to selectively bind and fuse with homologous BC exosomes, enabling precise molecular subtyping of BC. The system detects endogenous exosomal RNA biomarkers (e.g., miR-375 for ER-positive and PD-L1 mRNA for triple-negative subtypes) to trigger catalytic DNA assembly, amplifying electrochemical signals for highly sensitive detection with a limit of 557 particles/mL. Key benefits also encompass high specificity and sensitivity, the option for non-invasive detection through liquid biopsy, the ability to monitor disease according to its stage, and versatility for multiple cancer types, positioning it as a powerful solution for BC diagnosis and personalized treatment planning 141.

**Ovarian cancer (OC)** is a leading cause of gynecological cancer-related deaths, often asymptomatic in early stages, earning it the nickname "silent killer." It primarily originates from epithelial cells but can also arise from germ cells or stromal tissues. Symptoms like abdominal bloating and pelvic pain are nonspecific, leading to delayed diagnosis. Risk factors include genetic predisposition (e.g., BRCA1/2 mutations), family history, age, and hormonal influences. Despite treatment advances, prognosis for advanced-stage cases remains poor due to high recurrence rates 142. Current diagnostic tools, including imaging, CA-125 and HE4 blood tests, are limited by low sensitivity and specificity, often resulting in false positives or negatives, especially for early-stage detection 143. CA125 and HE4 are the only approved biomarkers for use in epithelial OC; however, they are not sufficient for early detection. Multivariate index (MVI) assays have been developed to mitigate the limitations of single serum biomarkers in epithelial OC, especially during the pre-surgical evaluation of adnexal masses. The Risk of malignancy algorithm (ROMA) integrates menopausal status, CA125 and HE4 concentrations to diagnose women with a pelvic mass. miRNAs may have remarkable potential in various aspects of epithelial OC prediction. However, further work is needed regarding its characterization as a biomarker. In particular, the steps involved in processing samples need to be standardized and the platforms for detecting miRNA in tumours and blood need to be refined 144. Invasive procedures like biopsies are costly and risky. Exosomal biomarkers offer a promising non-invasive alternative for early OC detection 145.

**Figure 5:**
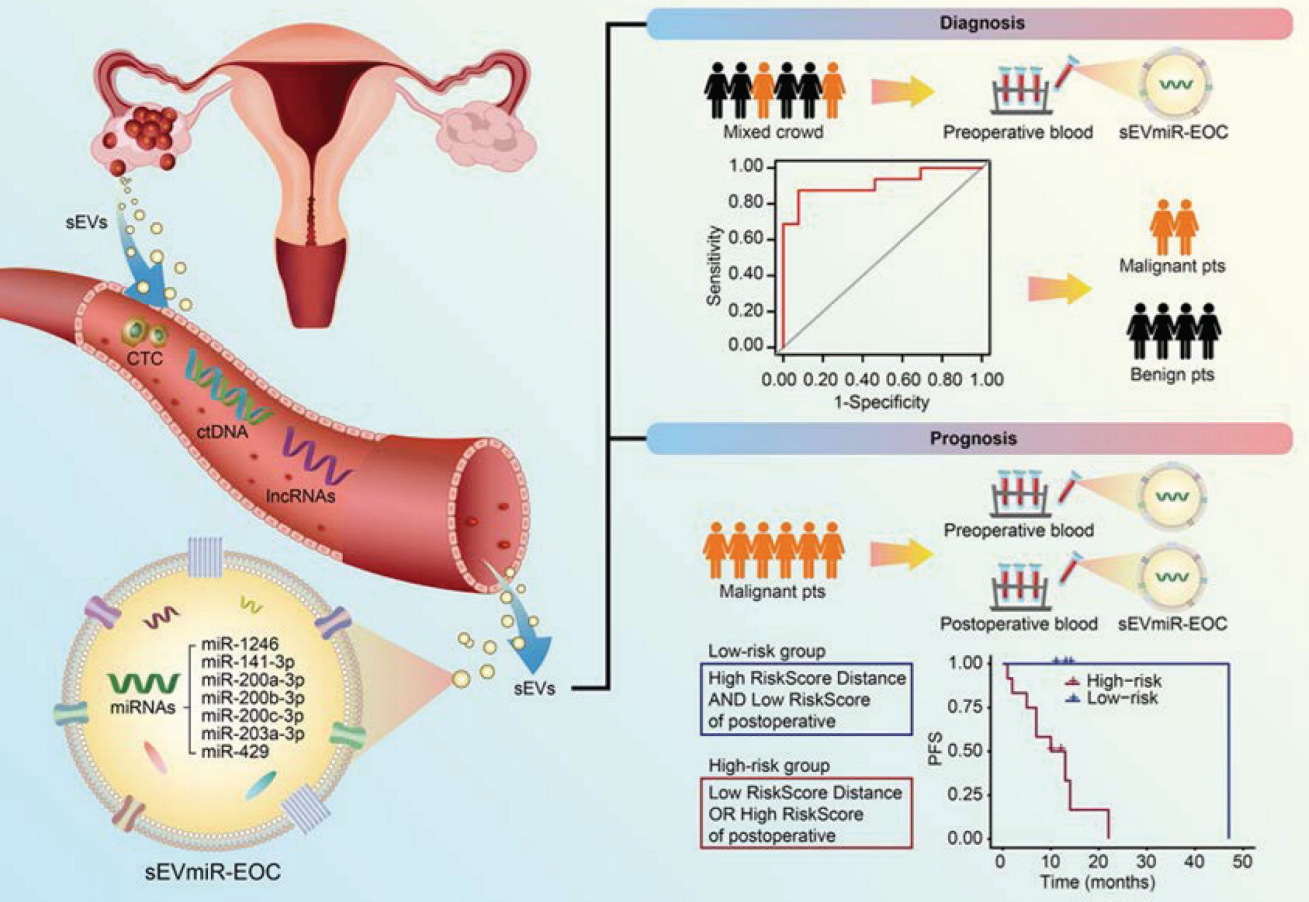
Schematic representation of the sEVmiR-EOC model for noninvasive diagnostic and prognostic predictions of EOC. The changes in the sEVmiR-EOC RiskScore between preoperative and postoperative blood samples were analyzed, and we found that the sEVmiR-EOC model could predict the prognosis of EOC patients. The study indicates that serum sEV miRNAs could be promising noninvasive biomarkers for the diagnosis and prognosis of EOC. Reproduced from reference 148 Copyright 2023, with permission from American Chemical Society.

Yanlong Xing *et al.* developed a SERS-microfluidic platform (S-MMEV) for non-invasive phenotyping of sEVs in serum, enabling early-stage OC diagnosis. The system combines SERS with microfluidics to detect multiple biomarkers, such as EpCAM, CD24, and CA125, with a detection limit as low as 10 particles/mL. By capturing sEVs with anti-CD63 antibodies and using SERS nanoprobes for biomarker labeling, it achieves high sensitivity and specificity (AUC: 0.9467 for early-stage detection). Its advantages include rapid turnaround (within one hour), the ability to detect multiple biomarkers simultaneously, non-invasive sample collection via liquid biopsy, and potential adaptability to various cancer types, making it a valuable tool for early detection and treatment monitoring 146. Genxi Li *et al.* developed an electrochemical biosensor based on entropy-driven autocatalytic DNA circuits (EADC) for the ultra-sensitive detection of ovarian cancer-derived exosomes. The biosensor captures exosomes expressing MUC1 glycoprotein using aptamer-functionalized probes, triggering an autocatalytic DNAzyme reaction that amplifies electrochemical signals. It achieved an impressive detection limit of 30 particles/μL and demonstrated high specificity in distinguishing OC exosomes from normal cell-derived exosomes. Strengths include its high sensitivity, non-invasive detection via serum samples, cost-effectiveness due to its enzyme-free design, adaptability for other biomarkers, and potential for portable, real-time cancer diagnostics, making it a powerful tool for early ovarian cancer detection 147. Zhiyong Liang *et al.* developed the sEVmiR-EOC model, a non-invasive diagnostic and prognostic tool for epithelial ovarian carcinoma (EOC) based on seven serum-derived sEV microRNAs (e.g., miR-141-3p, miR-200c-3p). This model achieved superior diagnostic accuracy (AUC: 0.913-0.973) compared to CA125, particularly in detecting early-stage EOC (AUC: 0.903 vs. 0.694). It also demonstrated prognostic utility by correlating miRNA expression changes with patient outcomes. The approach provides high sensitivity, specificity, and the capability to track disease progression, making it a promising method for early detection, treatment monitoring, and personalized management of OC, with potential adaptability for other types of cancer 148 (Figure 5).

**Renal cell carcinoma (RCC)** is the most common kidney cancer, accounting for about 90% of adult cases. It originates in the renal tubules and is known for its aggressive nature and resistance to traditional therapies like chemotherapy and radiotherapy 149. Early-stage RCC is often asymptomatic, while advanced stages may present with hematuria, flank pain, and an abdominal mass. Risk factors include smoking, obesity, hypertension, and genetic factors like mutations in the VHL tumor suppressor gene. The loss of functional VHL protein is considered a defining event in the development of clear-cell RCC, and the principal downstream oncogenic mechanism appears to be HIF-2α accumulation and constitutive HIF transcription factor activity. Belzutifan is a potent small-molecule inhibitor of HIF-2α that prevents heterodimerization with HIF-1β into an active transcription factor and has shown activity in clear-cell RCC. A recently published phase III study showed a significant benefit of belzutifan over everolimus concerning progression-free survival and objective response in participants with advanced clear-cell RCC who had previously received immune checkpoint and antiangiogenic therapies 150. Diagnosis faces challenges, as RCC is frequently detected incidentally and current imaging methods (CT, MRI, ultrasound) may not distinguish benign from malignant lesions, often requiring invasive biopsies for confirmation 151. The heterogeneity of RCC further complicates diagnosis, emphasizing the need for advanced diagnostic tools, including molecular biomarkers and improved imaging methods for early, non-invasive detection. The cargo in tumor-derived exosomes, such as the range of miRNAs, can serve as biomarkers for clear cell RCC in the serum and urine of patients, offering valuable targets for early detection and monitoring of the disease 152.

Fubo Wang *et al.* identified five exosomal mRNA biomarkers (CUL9, KMT2D, PBRM1, PREX2, SETD2) for early detection and differential diagnosis of clear cell renal cell carcinoma (ccRCC). The diagnostic signature with KMT2D and PREX2 achieved high accuracy (AUC: 0.836-0.830), and the differential signature with CUL9, KMT2D, and PREX2 distinguished ccRCC from benign renal masses (AUC: 0.816). This approach, validated across multiple cohorts, offers exceptional sensitivity, specificity, and non-invasive detection using blood-derived exosomes, making it a valuable tool for early ccRCC detection and personalized treatment 153. Maria Jesus Alvarez-Cubero *et al.* identified exosomal mitochondrial DNA (mtDNA) as a non-invasive biomarker for RCC diagnosis and aggressiveness. mtDNA genes like HV1 and CYB showed significant differences between RCC patients and healthy controls, correlating with tumor stage and metastatic potential. Using plasma-derived exosomes and digital PCR, the study achieved high diagnostic accuracy (AUC: 0.833 for HV1), demonstrating the stability of exosomal mtDNA in circulation and its value for monitoring RCC progression and guiding personalized treatments 154. Kerstin Junker *et al.* identified CD147, CA9, and CD70 as specific tumor markers on exosomes from ccRCC, distinguishing them from normal tissue exosomes. These markers, validated in ccRCC cell lines, primary tumors, and patient samples, reflect key tumorigenic processes like matrix degradation, angiogenesis, metabolism, and immune evasion. The study introduced a refined EV isolation protocol for high-purity EV recovery, showing that these markers outperform EpCAM in diagnostic performance and have potential for non-invasive early diagnosis and therapeutic monitoring of ccRCC 155.

The studies highlight significant advancements in non-invasive diagnostics for urogenital cancers (Table 7). Exosomal biomarkers such as miR-2276-5p (OC), miR-141-3p (RCC), and tumor-specific surface markers (e.g., CD147, CA9, CD70) are emerging as key diagnostic tools. Advanced detection technologies, including SERS-based biosensors, CRISPR/Cas12a assays, and autocatalytic DNA circuits, enable highly sensitive and specific detection of these biomarkers in liquid biopsy samples. With diagnostic accuracies (AUC > 0.9), these platforms allow early cancer detection, differentiation of malignant and benign conditions, and precise molecular subtyping. They also support disease monitoring and prognostic assessments, crucial for personalized treatment. Novel isolation and amplification methods ensure reproducibility, making these approaches clinically feasible. These innovations represent a major shift in urogenital cancer diagnostics, improving early detection, prognosis, and treatment outcomes.

### 4.7. Dermal system

**Malignant melanoma (MM)** is an aggressive skin cancer originating from melanocytes, responsible for most skin cancer-related deaths due to its rapid metastasis. Risk factors include UV exposure, fair skin, sunburns, and genetic mutations (e.g., BRAF, NRAS) 156. Early symptoms involve changes in moles, following the ABCDE rule. Diagnosis faces challenges as early-stage melanomas resemble benign lesions, and visual assessments may miss atypical cases. Dermoscopy and biopsy are the gold standards but are invasive and require expertise. Advanced melanomas often metastasize early, and the lack of specific biomarkers complicates non-invasive screening 157. Myeloid-derived suppressor cells, Treg cells and tumour-associated macrophages constitute immunosuppressive cells present within the tumour microenvironment, which release ROS amongst other factors, effectively inhibiting NK cell response. Higher levels of fibroblasts secrete more metalloproteinases, resulting in further shedding of ligands that could link to NK cells. Fibroblasts even have a more direct impact on NK cells by preventing cytokine induced activating receptor upregulation. NK cell function is also impaired by the presence of melanoma derived exosomes. This finding highlights the potential of exosomal biomarkers in diagnostics 158.

Se-Hwan Paek *et al.* developed a calcium switch-based immuno-isolation method to enrich CD63-positive exosomes for early melanoma detection. This approach increased diagnostic sensitivity (87.5%) compared to bulk exosome analysis (68%) and accurately distinguished melanoma patients from healthy controls (specificity: 85.7%). The method offers a non-invasive, reliable alternative for cancer detection and can be applied to other cancers 159. Amalia Azzariti *et al.* identified PD1+ and PD-L1+ EVs as biomarkers for predicting response to immune checkpoint inhibitors (ICIs) in metastatic melanoma. High levels of these EVs correlated with poorer prognosis and contributed to resistance by neutralizing therapeutic antibodies and suppressing T-cell activity. Liquid biopsy of circulating EVs offers a predictive, minimally invasive method for monitoring ICI response 160. Cong Peng *et al.* found that plasma exosomal miR-1180-3p is a promising non-invasive biomarker for cutaneous melanoma, with an AUC of 0.729. miR-1180-3p was downregulated in melanoma patients and suppressed tumor cell proliferation and migration by targeting key genes involved in melanoma progression. This highlights its potential for early detection and personalized treatment 161.

**Non-melanoma skin cancer (NMSC)**, the most common cancer worldwide, primarily includes basal cell carcinoma (BCC) and squamous cell carcinoma (SCC) 162. BCC is generally slow-growing with minimal metastatic risk, while SCC has a higher potential for invasion and metastasis. Key risk factors include chronic UV radiation exposure, fair skin, immunosuppression, and genetic predispositions like xeroderma pigmentosum 163. NMSC typically manifests as non-healing lesions or erythematous plaques on sun-exposed areas, with BCC appearing as pearly nodules and SCC as scaly, erythematous lesions 164. Diagnosis of non-melanoma skin cancer (NMSC) relies on clinical examination and biopsy, with treatments ranging from surgery (e.g., Mohs) to cryotherapy, radiation, and topical therapies. Advanced cases may require systemic immunotherapy, such as cemiplimab. Early intervention offers an excellent prognosis, while UV protection and regular skin checks are key to managing the increasing global incidence of NMSC. Diagnosis faces limitations, especially in early detection and differentiation from benign conditions. Clinical examination and dermoscopy depend on clinician experience and may miss subtle presentations, particularly in early-stage tumors or patients with darker skin. Biopsy is invasive and prone to sampling errors. Emerging imaging techniques, like ultrasound and reflectance confocal microscopy, are not widely available. There is also a lack of reliable non-invasive biomarkers for distinguishing aggressive subtypes. These challenges can lead to delayed or misdirected treatment. Advancements in non-invasive diagnostics, such as liquid biopsy, molecular profiling, and AI-assisted imaging, are needed to improve early detection and accuracy 165.

A.L.S. Chang *et al.* identify serum exosomal miR-197-5p as a key regulator of metastasis in BCC, highlighting its role in modulating the tumor microenvironment. RNA sequencing and qPCR revealed that miR-197-5p, among other miRNAs, was significantly upregulated in serum exosomes from metastatic BCC (MBCC) patients compared to non-metastatic cases. Functional studies demonstrated that MBCC-derived exosomes enhanced fibroblast proliferation, migration, and invasion, with miR-197-5p specifically promoting fibroblast activation. Inhibition of miR-197-5p significantly reduced fibroblast migration and metabolic activity, underscoring its role in metastasis. These findings imply that miR-197-5p could serve as a non-invasive biomarker for metastatic BCC and a potential therapeutic target, pending further validation in larger clinical studies 166. Jiuhong Li *et al.* identify exosomal circ-CYP24A1 as a critical driver of cutaneous squamous cell carcinoma (cSCC) progression and a potential diagnostic biomarker and therapeutic target. circ-CYP24A1 was significantly overexpressed in plasma-derived exosomes from cSCC patients and correlated with tumor size, thickness, and serum SCC-Ag levels. Functional studies revealed that circ-CYP24A1 promotes cSCC cell proliferation, migration, and invasion while inhibiting apoptosis, partially through the regulation of downstream targets such as CDS2, MAVS, and SOGA1. Pathway analysis linked circ-CYP24A1 to immune and cell cycle regulatory pathways, highlighting its role in tumor-stromal communication. Targeting circ-CYP24A1 in exosomes significantly reduced tumorigenic behaviors, underscoring its potential as a non-invasive biomarker for cSCC diagnosis and a novel therapeutic target for limiting tumor progression 167.

Exosomes are emerging as highly valuable tools in the diagnosis and monitoring of skin cancers, including basal cell carcinoma, malignant melanoma, and squamous cell carcinoma (Table 8). By carrying tumor-specific biomarkers such as CD63, Cav1, circ-CYP24A1, and miR-1180-3p, exosomes enable highly sensitive and specific detection of early-stage cancers and offer insights into disease progression. Moreover, exosomal molecules like PD1 and PD-L1 are closely linked to immunotherapy resistance and poor prognosis in metastatic melanoma, making them effective for predicting therapeutic outcomes and enabling real-time monitoring of treatment efficacy. Additionally, exosomal miRNAs such as miR-197-5p play a role in metastatic progression, highlighting their potential as therapeutic targets. With their high stability in circulation, non-invasive collection from bodily fluids, and ability to provide dynamic molecular insights, exosomes represent an ideal platform for liquid biopsy, offering transformative opportunities for early detection, personalized treatment, and improved patient management in skin cancers.

### 4.8. Motor system

**Osteosarcoma,** the most common primary malignant bone tumor, primarily affects adolescents and young adults. It arises from mesenchymal cells and commonly occurs in the long bones, especially near the metaphysis of the distal femur, proximal tibia, and humerus 168. Risk factors include genetic mutations (e.g., RB1, TP53), Paget's disease, and radiation exposure. Symptoms, such as localized pain and swelling, often worsen over time. Treatment involves chemotherapy and surgical resection, with limb-sparing surgeries improving quality of life. However, metastasis and recurrence make it a challenging disease 169. Early diagnosis is difficult, as initial symptoms are often nonspecific and can be mistaken for benign conditions. Current imaging techniques, while useful, cannot distinguish between benign and malignant lesions without biopsies, which are invasive and carry risks. Additionally, there are no established blood-based biomarkers for osteosarcoma, limiting early detection. These diagnostic challenges often lead to advanced-stage diagnoses when metastases are more common, highlighting the need for innovative, non-invasive diagnostic methods to improve early detection and outcomes.

Liang Qiao *et al.* developed a microfluidic-SERS platform for non-invasive osteosarcoma diagnosis by profiling plasma-derived exosomal biomarkers (CD63, VIM, EpCAM). The system combines tangential flow microfluidics with SERS nanotags, achieving high sensitivity (2 exosomes/μL) and diagnostic accuracy (sensitivity: 100%, specificity: 90%, overall accuracy: 95%). Requiring minimal plasma volume (50 µL) and delivering results in 5 hours, the platform is faster, more sensitive, and cost-effective compared to traditional methods. Its ability to detect multiple biomarkers simultaneously offers significant potential for early osteosarcoma detection and personalized treatment planning 170. Amos HP Loh *et al.* identified exosomal mRNA biomarkers (THBS1, MS4A1, TCL1A) from peripheral blood as non-invasive indicators of tumor burden, immune response, and minimal residual disease (MRD) in pediatric osteosarcoma. These biomarkers correlate with disease progression and treatment response, with exosomal mRNA outperforming circulating tumor cells (CTCs) in reliability and RNA integrity for monitoring disease dynamics. This advancement offers potential for real-time, minimally invasive monitoring and personalized treatment strategies 171. Jinbo Liu *et al.* identified plasma exosome-derived SENP1 as a highly accurate biomarker for prognosis and monitoring of osteosarcoma. SENP1 levels were elevated in osteosarcoma patients and correlated with clinical features like tumor size, stage, and metastasis. The study showed high prognostic accuracy with AUCs of 0.96 for 3-year DFS and OS. SENP1 levels also decreased following treatment, emphasizing its role in real-time monitoring. These findings highlight the potential of plasma exosome-derived SENP1 for early intervention, personalized treatment, and improved prognostic accuracy in osteosarcoma 172.

Exosome biomarkers like SENP1, miR-335-5p, miR-1246, and CD147 are valuable tools for diagnosing, prognosing, and managing musculoskeletal tumors (Table 9). Found in plasma-derived exosomes, they offer high sensitivity and specificity for tumor detection. SENP1 shows strong prognostic value in osteosarcoma (AUROC: 0.90 for 1-year, 0.96 for 3-year survival). Exosomal biomarkers enable non-invasive early detection, monitoring, and provide insights into tumor biology, such as promoting metastasis and tumor-stroma interaction. They hold promise for personalized oncology, improving diagnostics, prognosis, and therapy.

## 5. Clinical feasibility and challenges

Exosomes have emerged as promising biomarkers for diagnosis, garnering significant attention in recent years. Considerable progress has been made through ongoing clinical trials. Studies increasingly demonstrate the unique advantages of exosomes in early detection, disease monitoring, and treatment evaluation, positioning them as strong candidates for non-invasive diagnostic tools. Currently, numerous clinical trials are underway to validate the efficacy and reliability of exosome-based diagnostics across various tumors 173. We have compiled clinical trial data in Table 10, covering biomarkers (protein and RNA, etc.) for detecting various cancers such as gastric, thyroid, colorectal, lung, bladder, kidney, lymphoma, pancreatic, ovarian, sarcoma, osteosarcoma, cholangiocarcinoma, melanoma, and so on. Several of these clinical trials have already shown the effectiveness of sEVs or exosomes as biomarkers.

A study developed a liquid biopsy method using the lncRNA GClnc1 derived from exosomes for early gastric cancer (EGC) diagnosis. Involving 2,141 participants, GClnc1 was significantly upregulated in gastric cancer tissues and circulating exosomes, outperforming traditional biomarkers (CEA, CA72-4, CA19-9) with high sensitivity (>85%) and specificity (>78%) in validation cohorts. It distinguished precancerous lesions from gastric cancer and identified patients negative for traditional biomarkers. Its levels decreased post-surgery, confirming specificity, and it was stable under conditions like room temperature and freeze-thaw cycles, making it a reliable tool for early detection and curative surgery (NCT05397548) 174. Further advancements in cancer detection include the DESTINEX trial, focused on exosome-based diagnostics for gastric cancer, which was completed (NCT06342427). This study aims to develop a cost-effective blood test for early gastric cancer detection by combining genetic markers, such as cell-free and exosomal microRNAs, to enhance accuracy. This approach could significantly reduce gastric cancer mortality and introduce new screening methods 175. A study developed the ExoVita Pancreas blood-based EV classifier for early detection of pancreatic ductal adenocarcinoma (PDAC). Analyzing seven protein biomarkers in EVs isolated from plasma, the classifier achieved high sensitivity (93.3%) and specificity (91.0%) in training and 90.0% sensitivity and 92.8% specificity in independent validation. It outperformed traditional markers like CA19-9, demonstrating its potential for early-stage PDAC detection and high-risk population screening. Ongoing investigations include the ExoLuminate Study (NCT0562552) to evaluate this method in high-risk PDAC patients 176. Lin Miao *et al.* identified bile-derived exosomal miR-483-5p and miR-126-3p as biomarkers for distinguishing malignant from benign biliary obstructions. RNA sequencing in 82 patients showed significant elevation of both miRNAs in malignant cases. miR-483-5p had an AUC of 0.81 (81.1% sensitivity and specificity), while miR-126-3p had an AUC of 0.74 (73.0% sensitivity, 86.5% specificity), outperforming CA19-9. These findings highlight the potential of miR-483-5p and miR-126-3p as effective, non-invasive diagnostic tools for malignant biliary obstructions (NCT03102268) 177. The GeparSixto clinical trial demonstrated the potential of exosomal miRNAs, particularly miR-155 and miR-301, as non-invasive biomarkers for tumor diagnostics. Specifically, miR-155 levels were significantly associated with pathological complete response (pCR) in both HER2-positive and triple-negative breast cancer (TNBC) patients, with a p-value of 0.002 in univariate and 0.003 in multivariate models. Similarly, miR-301 showed a strong correlation with pCR, with p-values of 0.002 in univariate and 0.001 in multivariate analyses. The study found that these biomarkers could potentially predict the therapeutic response to neoadjuvant therapy, particularly in the carboplatin treatment arm. However, further studies are required to validate these biomarkers in larger cohorts and to improve exosome isolation methods, as current exosome populations are heterogeneous, containing both cancerous and normal exosomes 178. One study on exosomal HOTTIP as a potential diagnostic and prognostic biomarker for GC found that exosomal HOTTIP levels were significantly elevated in GC patients compared to healthy controls, with a p-value of <0.001. The diagnostic capability of exosomal HOTTIP was superior to traditional markers such as CEA, CA 19-9, and CA 72-4, with an AUC of 0.827, compared to 0.653, 0.685, and 0.639 for the latter, respectively. Additionally, high levels of exosomal HOTTIP were correlated with worse OS, with Kaplan-Meier analysis indicating a significant association (logrank P < 0.001). Multivariate Cox regression confirmed that exosomal HOTTIP was an independent prognostic factor for poor OS in GC patients (P = 0.027). These findings suggest that exosomal HOTTIP could serve as a valuable non-invasive biomarker for both diagnosing and predicting the prognosis of gastric cancer 179.

Although exosomal biomarkers have shown promising results in clinical trials, several challenges still hinder their further development and application. Firstly, the isolation and purification of exosomes remain challenging, as current methods often lack specificity and scalability, leading to contaminants and inconsistent results 180. Secondly, the absence of standardized protocols for isolation, quantification, and characterization creates reproducibility issues across studies. Additionally, the heterogeneity of exosomes complicates biomarker identification, while existing detection methods lack the sensitivity required for low-abundance biomarkers 181. Furthermore, the limited understanding of exosomal biogenesis and cargo selection mechanisms hinders data interpretation 182. Moreover, scalability issues for clinical applications and regulatory hurdles for biomarker validation pose significant barriers. Finally, biological variability among patients and limited access to large clinical sample cohorts further complicate biomarker discovery and validation. Addressing these challenges requires integrated advancements in technology, biology, and clinical frameworks to fully realize the potential of exosomal biomarkers 183.

## 6. Future directions and opportunities

The future of exosome-based diagnostics is poised for significant advancement, with several promising directions paving the way for its development. The integration of AI and machine learning (ML) offers the potential to enhance data analysis, enabling greater diagnostic accuracy, predictive modeling, and the ability to identify novel biomarker patterns from large, complex datasets 184. Exosomal biomarkers hold significant promise in personalized medicine and precision diagnostics, providing opportunities for tailored disease management, early intervention, and improved patient outcomes. Beyond diagnostics, exosome research is expanding into therapeutic applications, including drug delivery systems and targeted gene therapies, further highlighting their clinical versatility. To fully realize their clinical utility, large-scale, multicenter studies are essential for validating these biomarkers across diverse populations, ensuring reproducibility, and establishing standardized protocols for exosome isolation, characterization, and biomarker quantification 185. Fubo Wang *et al.* discovered that by analyzing tumor RNA derived from exosomes, a set of blood-based biomarkers for multi-cancer early detection was identified. These biomarkers were validated through a multi-phase, multi-center study and used to develop a machine learning-based diagnostic platform (ETR.sig). This platform efficiently distinguishes cancer from healthy controls, with an AUC of 0.915, and accurately classifies multiple cancer types, with a multi-class model achieving an AUC of 0.983. The study also demonstrated that these exosomal RNA biomarkers are closely associated with tumor progression and prognosis, with higher ETR.sig scores correlating with advanced cancer stages and poorer survival outcomes 186. Furthermore, leveraging advances in microfluidics and biosensor technology could streamline exosome analysis, making it faster, more cost-effective, and accessible for routine clinical use 187. The iExoDisc, developed by Xudong Zhao and colleagues, is an automated centrifugal microfluidic platform for efficiently isolating exosomes from blood samples and performing glycan analysis. The benefits of this platform include: completing exosome isolation within 45 minutes, saving significant time compared to traditional methods like ultracentrifugation; improving exosome purity by 3 to 6 times and achieving a recovery rate of 74.7%; additionally, iExoDisc can identify potential diagnostic markers, such as galactosylation and sialylation, from plasma samples of triple-negative breast cancer (TNBC) patients. This technology provides a new solution for early cancer diagnosis and liquid biopsy 188. Exploring their application in new disease areas, such as cardiovascular, metabolic, autoimmune disorders, and even rare genetic diseases, could vastly expand their impact and provide novel insights into disease pathogenesis 189. Additionally, future research should focus on exploring multi-biomarker analysis and combined diagnostic strategies to enhance both the sensitivity and specificity of diagnostics. The performance of a single biomarker may be limited, but integrating various exosomal RNAs, proteins, and lipids can provide a more comprehensive view of the multifaceted nature of disease states. Such multi-biomarker combinations are expected to offer more reliable tools for risk stratification and disease classification, ultimately enabling more precise disease management. Moreover, developing multimodal diagnostic approaches that combine exosomal biomarkers with other diagnostic technologies, such as imaging and immunoassays, could further improve diagnostic comprehensiveness and accuracy 190. Technological advancements will continue to drive the evolution of the exosome field. Emerging nanotechnologies, enhanced isolation techniques, and high-throughput analytical platforms are poised to significantly reduce the time and cost of exosome detection. At the same time, the development of miniaturized, portable diagnostic devices--such as microchips for bedside testing--could bring exosome diagnostics directly into clinical practice, particularly in resource-limited settings. Furthermore, optimizing the engineering of exosomes could lead to the production of specific exosome mimics or "smart exosomes" that more precisely deliver diagnostic and therapeutic information, thereby providing more targeted solutions for future personalized medicine 191. From a regulatory and policy perspective, the future of exosome diagnostics and therapeutics will benefit from more clearly defined regulatory frameworks and industry guidelines. Establishing well-defined regulations and industry standards can reduce uncertainties in the development process, accelerate the approval of new products, and build trust among the public and medical community. Collaboration between industry and regulatory agencies will be critical for developing unified quality benchmarks, validation standards, and safety evaluation protocols 5, 192. Finally, patient engagement and education will be crucial to advancing exosome research and clinical applications. Increasing public awareness of exosome technologies and their potential not only enhances patient acceptance of exosome-based diagnostics and therapies, but also encourages greater participation in clinical trials, providing richer datasets for research. Strengthening medical education, including helping clinicians understand how to interpret exosomal biomarkers and integrate them into existing clinical pathways, will also facilitate the widespread adoption and application of this technology in routine clinical practice.

Addressing these opportunities will help overcome current challenges, such as variability in isolation techniques, limited scalability, and a lack of universally accepted biomarkers. By resolving these hurdles and fostering interdisciplinary collaboration, the transformative potential of exosomal biomarkers in advancing diagnostics, therapeutic monitoring, and patient care can be fully unlocked, marking a new era in precision medicine.

## 7. Conclusion

Exosomal biomarkers have the potential to fundamentally transform the landscape of non-invasive cancer diagnostics, offering a highly promising alternative to traditional invasive methods. These nanoscale vesicles, carrying a rich cargo of proteins, lipids, RNA, and microRNAs, provide a snapshot of the molecular state of their parent cells, reflecting critical changes that occur during disease progression. As such, exosomes represent an unparalleled tool for detecting cancer at early stages, monitoring tumor dynamics, and assessing treatment responses in real time. One of the most compelling advantages of exosome-based diagnostics is their ability to be extracted from easily accessible biofluids such as blood, saliva, and urine, positioning them as ideal candidates for liquid biopsy technologies. The ability to perform repeated, non-invasive sampling makes exosomes particularly attractive for continuous monitoring of tumor progression and therapeutic efficacy, significantly improving patient outcomes. Recent advancements in exosome isolation and characterization techniques have substantially enhanced their sensitivity and specificity, allowing for the detection of tumor-specific biomarkers even at low concentrations in biofluids. Clinical trials incorporating exosome-based diagnostics have demonstrated their utility across various cancer types, from respiratory and digestive system cancers to hematological and neurological malignancies. These trials have showcased exosomes as reliable biomarkers for early cancer detection, offering diagnostic accuracy on par with or exceeding traditional methods such as tissue biopsies and imaging. Additionally, exosome-based diagnostics offer significant advantages in terms of their ability to capture tumor heterogeneity, providing a comprehensive picture of disease status, which is often challenging to achieve through conventional approaches. Despite these exciting developments, several challenges remain in fully realizing the potential of exosome-based diagnostics. The biological heterogeneity of exosomes, both within and between different diseases, poses a significant obstacle in identifying universal biomarkers with sufficient specificity. Furthermore, the deficiency of standardized guidelines for exosome isolation, characterization, and analysis continues to hinder the widespread implementation of these technologies in clinical settings. Regulatory hurdles, such as the need for clinical validation and approval by regulatory bodies, also represent significant barriers to the clinical adoption of exosome-based diagnostics. Nevertheless, the future of exosome-based diagnostics remains incredibly promising. With continued advancements in exosome isolation methods, the development of more refined and standardized analytical techniques, and the successful completion of large-scale clinical trials, exosomes are poised to revolutionize the field of personalized medicine. Their ability to provide dynamic, real-time insights into tumor behavior and response to treatment could significantly enhance the precision and effectiveness of cancer therapy. As exosome-based technologies mature, they are expected to play a central role in early cancer detection, improving patient prognosis through earlier interventions, better monitoring of disease progression, and more tailored therapeutic strategies.

Ultimately, integrating exosomal biomarkers into routine clinical practice will offer a paradigm shift in cancer diagnostics and management. It will empower clinicians with powerful tools for personalized treatment, enable more effective and less invasive monitoring, and enhance patient outcomes by allowing for timely intervention. With the field undergoing further evolution, exosomes are positioned to play a transformative role in cancer care, providing promise for enhanced accuracy, cost-effective, and accessible diagnostic solutions.

## Figures and Tables

**Table 1:** Methods for exosome-based diagnostic purification and detection

Method	Type	Strengths	Limitations
Ultracentrifugation	Purification	Cost-effective, widely used	Time-consuming, low yield, may co-isolate contaminants
Size Exclusion Chromatography	Purification	High purity, gentle process	Lower throughput, requires specialized equipment
Immunocapture	Purification	High specificity, rapid isolation	Potential bias toward targeted subpopulations
Precipitation Kits	Purification	Fast, cost-effective	Lower purity, co-precipitation of contaminants
Fluorescence-Based Techniques	Detection	High sensitivity, suitable for real-time analysis	Requires fluorescent labels, complex assay setup
Nanoparticle Tracking Analysis	Detection	Quantifies size and concentration	Lacks specificity for surface markers
Western Blotting	Detection	Identifies specific proteins or biomarkers	Requires large sample volumes, time-consuming
Enzyme-Linked Immunosorbent Assay	Detection	High sensitivity for protein biomarkers	Time-consuming, requires prior exosome isolation
Surface-Enhanced Raman Spectroscopy	Detection	High sensitivity, label-free, multiplexing capabilities	Complex sample preparation, requires specialized equipment
Electrochemical Sensors	Detection	Low-cost, sensitive, suitable for point-of-care testing	Requires electrode-based setups, limited to specific biomarkers

**Table 2:** Exosomal biomarkers in respiratory system cancers

Cancer type	Biomarkers	Source	Analytical Technique	Expression	Effectiveness	Reference
	miR-155	Plasma	Integrated concentration and determination system of exosomes (ICDSE)	Upregulated	LOD: 2.03 fM; Specific & Cost-effective	59
Lung cancer	YES1	Plasma	Western blot, IHC, FISH	Upregulated	Independent predictor of poor prognosis, correlates with reduced PFS and OS	60
	Autoantibody panel on sEVs (P53, PGP9.5, SOX2, GAGE7, GBU4-5, MAGEA1, CAGE)	Plasma	ELISA, AUC analysis	Upregulated	Sensitivity: 65.56%, Specificity: 96.88%, AUC: 0.8007	61
Multi-cancer types (lung, breast, colon, liver, pancreas, stomach)	Raman signal patterns of exosomes	Plasma	Surface-enhanced Raman spectroscopy (SERS) with AI	Differential patterns of Raman signals	AUC: 0.970; Sensitivity: 90.2%; Specificity: 94.4%; Tissue of origin (TOO) accuracy: 72%	62
Nasopharyngeal carcinoma	hsa-miR-1301-3p	Plasma	RNA sequencing, qRT-PCR, GO/KEGG analysis	Upregulated	Differential expression confirmed (Fold Change > 2, P < 0.05); GO/KEGG pathways indicate roles in PI3K-Akt and MAPK pathways	66
	miR-24-3p	Serum	RT-qPCR, Luciferase reporter assay, FACS analysis	Upregulated	Elevated serum miR-24-3p levels link to poor DFS (p<0.05) and immune regulation.	67
	circMYC	Serum	qRT-PCR, AUROC analysis, siRNA knockdown studies	Upregulated in radioresistant NPC	AUROC: 0.945 Sensitivity: 90.24%, Specificity: 94.51%	68

**Table 3:** Exosomal biomarkers in digestive system cancers

Cancer type	Biomarkers	Source	Analytical Technique	Expression	Effectiveness	Reference
	miRNA-135b	Ascites	MoS2 QDs-MXene-based ECL sensor, qRT-PCR	Upregulated	LOD: 10 fM; Linear range: 30 fM-20 nM; High specificity and sensitivity for GC diagnosis	72
Gastric cancer	MUC1	Ascites	Magnetoplasmonic metasurface-modulated ECL sensor	Upregulated	Detection range: 5.8×10^2^ to 5.8×10^7^ particles/mL; LOD: 200 particles/mL; High specificity and stability	73
	MUC1, CD63	Serum	Droplet Digital Branched Rolling Circle Amplification (ddBRCA)	Upregulated	Detection range: 2×10^2^ to 3.2×10^3^ particles/mL; LOD: 12 particles/mL; AUC (MUC1): 0.8086; AUC (CD63): 0.7832; Combined AUC: 0.8705; Sensitivity: 82.35%; Specificity: 66.67%	74
	piR-1029, piR-15254, novel-piR-35395, novel-piR-32132, novel-piR-43597	Serum	sRNA-seq, qRT-PCR, AUROC analysis	Upregulated	AUROC: 0.986 (combined); High sensitivity (82.35%) and specificity (66.67%); Superior to AFP	78
Hepatocellular carcinoma	lncRNA THEMIS2-211, PRKACA-202, H19-204	Plasma	qRT-PCR, AUROC analysis analysis	Upregulated	AUROC: THEMIS2-211 (0.832), PRKACA-202 (0.804), H19-204 (0.701); Combined AUROC: 0.879; Superior to AFP for early-stage HCC	79
	lncRNA LUCAT-1, EGFR-AS1	Plasma	SiO₂-chip for exosome enrichment, qRT-PCR, AUROC analysis	Upregulated	Combined AUROC: 0.897 (LUCAT-1 + EGFR-AS1); Improved diagnostic accuracy when combined with AFP and DCP	80
	GPC1, EphA2	Plasma	Nanoliquid Biopsy (nLB) with ICP-MS quantification	Upregulated	AUROC: 1.0 (Stage I/II PDAC vs NC); LOD: 78 pg/mL GPC1; Sensitivity and Specificity: 100%	83
Pancreatic cancer	miRNA-21, miRNA-16, miRNA-10b, miRNA-155, miRNA-1246, miRNA-196a	Plasma	Encoded fusion-mediated miRNA profiling with flow cytometry	Upregulated	AUROC: 0.98 (combined miRNAs for PDAC vs healthy donors); Sensitivity: 98%; Process time: 2 hours for multiplex detection	84
	GPC1 mRNA, GPC1 protein	Serum	Immune Lipoplex Nanoparticle (ILN) biochip, qRT-PCR, Western blot, TEM	Upregulated	AUROC (Stage I/II): 0.960 (dual-biomarker); AUROC (Stage III/IV): 0.973; Improved sensitivity and specificity compared to CA19-9	85
Colorectal cancer	circLPAR1	Plasma	RNA sequencing, FISH, qRT-PCR, RNA pull-down, RIP assay	Downregulated in CRC plasma and tissues; Upregulated after surgery	AUROC: 0.875 (combined with CEA and CA19-9); Sensitivity: 87.3%; Specificity: 76.3%; Correlates with tumor suppression and improved survival	91
	Panel of 10 protein markers (e.g., APOA4, GPX3, SNCA, THBS4, etc.)	Plasma	DSPE-functionalized bead isolation, SP3, DIA-MS, Machine Learning	Dysregulated proteins in early CRC and polyp patients	AUROC: 1.0 (SVM model); Accuracy: 89.3%; Sensitivity and specificity improved significantly	92
Colorectal cancer and advanced adenoma	10 RNA markers, including miR-425-5p, let-7f-5p, C19orf43, TOP1, PPDPF, lnc-MKRN2-42:1, HIST2H2AA4, and LNC-EV-9572	Plasma	RNA sequencing, qRT-PCR, t-SNE clustering, machine learning (SVM, Lasso regression)	Biomarkers showed distinct profiles in T1a CRC/AA patients compared to normal controls	AUROC: 0.88 for AA and 0.80 for CRC using multi-RNA signatures; Sensitivity: 99.0%, Specificity: 79.3% with RT-qPCR	93

**Table 4:** Exosomal biomarkers in hematologic system cancers

Cancer type	Biomarkers	Source	Analytical Technique	Expression	Effectiveness	Reference
	miR-221-3p	Bone marrow and plasma	RNA sequencing, qRT-PCR, flow cytometry, Western blot, TEM	Upregulated	Promotes leukemogenesis by inhibiting apoptosis and facilitating cell cycle entry; Targeting Gbp2 to regulate PI3K/AKT pathway	97
Leukemia	Nucleolin	Leukemia-derived exosomes	Rolling Circle Amplification (RCA) with dual signal amplification (colorimetric biosensor)	Upregulated	LOD: 100 particles/μL; High specificity; Successfully distinguishes leukemia patients from healthy individuals	98
	Exosomal lncRNAs (LINC00265, LINC00467, UCA1, SNHG1)	Plasma	qRT-PCR, AUROC analysis, TEM, NTA, Western blot	LINC00265, LINC00467, UCA1 downregulated; SNHG1 upregulated in AML patients compared to healthy donors	AUROC for combined biomarkers: 0.8685; sensitivity: 85%-100%; specificity: 50%-65%; effective in diagnosis and treatment monitoring	99
	miR-21	Bone marrow	qRT-PCR, Western blot, TEM, NTA	Upregulated	miR-21 promotes angiogenesis in MM by enhancing MMEC proliferation, migration, and tube formation; transforms NFs into CAFs	101
Multiple Myelom	circ-ATP10A	Serum	RNA-seq, qRT-PCR, TEM, NTA, Immunohistochemistry	Upregulated	AUROC: 0.854; Sensitivity: 87.5%; Specificity: 75%; Prognostic biomarker for angiogenesis and MM progression	102
	LRG1	Platelet	Proteomics, ELISA, qRT-PCR, Western blot, TEM, NTA	Upregulated	Promotes MM progression via EMT activation and angiogenesis; High LRG1 correlates with poor prognosis;	103
	miR-21-5p, miR-320e, miR-4454	Serum	Nanostring nCounter miRNA array, qRT-PCR, TEM, NTA, KEGG analysis	Upregulated	Predicts poor overall survival (OS); Associated with treatment failure; miR-21-5p (OS: p < 0.001); miR-4454 (OS: p < 0.001)	107
Lymphoma	miR-107	Plasma	qRT-PCR, RNA-seq, Western blot, TEM, NTA	Downregulated	AUROC: 0.854 for diagnostic power; inhibits cell proliferation and invasion by targeting 14-3-3η; strong prognostic relevance	108

**Table 5:** Exosomal biomarkers in nervous system cancers

Cancer type	Biomarkers	Source	Analytical Technique	Expression	Effectiveness	Reference
	miR-2276-5p	Plasma	qRT-PCR, AUROC analysis, TEM, NTA, western blot	Downregulated	AUROC: 0.8107; Prognostic biomarker for survival; Lower expression associated with poor survival rates	113
Glioma	miR-17-5p, miR-21-5p, miR-27a-3p, LOX, SLCO3A1	Plasma	EZ-READ platform, qRT-PCR, RNA sequencing, ROC analysis, TEM, NTA	Upregulated	AUROC for diagnosis: 0.854; AUROC for subtyping: 0.897; enables non-invasive subtype classification and prognosis	114
	CD63, CD44, CD133	Plasma	Multiplex optical biochip with nanochain patterns	Upregulated	Detects GBM-specific exosomes with high sensitivity (LOD: 6×10⁷ particles/mL); rapid detection (~30 min)	115
	circRNA BTG2, miR-25-3p, PTEN	Exosomes from RBP-J overexpressed macrophages	qRT-PCR, RNA-seq, TEM, NTA, Luciferase assay, Transwell	circRNA BTG2 upregulated; miR-25-3p downregulated; PTEN upregulated	Inhibits glioma progression; reduces proliferation and invasion via the circBTG2/miR-25-3p/PTEN pathway NFs into CAFs	116
	hsa-miR-486-5p, hsa-miR-151a-5p, hsa-miR-652-3p_R+1, hsa-miR-1180-3p	Serum	NGS, qRT-PCR, microarray, AUROC analysis, TEM, NTA, western blot	Upregulated	AUROC: 0.943 (hsa-miR-486-5p); potential biomarker for NFPA diagnosis, progression, and relapse prediction	119
Pituitary adenoma	circCCDC66	Serum	qRT-PCR, AUROC analysis, TEM, NTA, western blot	Upregulated	AUROC: 0.872; Sensitivity: 80%; Specificity: 84%; prognostic marker for tumor recurrence and progression	120
	N-cadherin, E-cadherin, Epcam, Vimentin	Serum	qRT-PCR, western blot, TEM, NTA, immunohistochemistry	N-cadherin and vimentin upregulated; E-cadherin and Epcam downregulated in PA	AUROC for N-cadherin/Epcam ratio: 0.83; effective in assessing tumor invasiveness and predicting recurrence	121

**Table 6:** Exosomal biomarkers in endocrine system cancers

Cancer type	Biomarkers	Source	Analytical Technique	Expression	Effectiveness	Reference
	miR-485-3p, miR-4433a-5p, miR-376a-3p	Plasma	Small RNA sequencing, qRT-PCR, AUROC analysis, TEM, NTA, Western blot	Upregulated	AUROC: miR-485-3p (0.858), miR-4433a-5p (0.812); serve as diagnostic and prognostic biomarkers for PTC	127
Thyroid cancer	Angiopoietin-1, TIMP	Urinary	LC-MRM/MS, qRT-PCR, AUROC analysis, TEM, NTA	Upregulated	AUROC: Angiopoietin-1 (0.857), TIMP (0.892); potential biomarkers for preoperative screening and prognosis	128
	miR-21-5p, miR-31-5p, miR-221-3p, miR-222-3p, let-7i-3p	Exosomes from thyroid cancer cell lines	qRT-PCR, RNA-seq, AUROC analysis, TEM, NTA, Western blot	Upregulated	AUROC: miR-31-5p (approximately 0.9), miR-222-3p (approximately 0.9); miRNAs linked to invasion, angiogenesis, and tumor progression	129
Pancreatic Neuroendocrine Neoplasm	miR-4488	Hypoxic tumor-derived exosomes	RNA-seq, qRT-PCR, TEM, NTA, WB, Luciferase assay	Upregulated	miR-4488 facilitates M2 macrophage polarization, promotes metastasis via RTN3/FABP5-mediated fatty acid oxidation and PI3K/AKT/mTOR pathway	134
	CEACAM5	Hypoxic tumor-derived exosomes	qRT-PCR, Western blot, ChIP, TEM, NTA, Transwell assay, Flow cytometry	Upregulated	AUROC: 0.873 for differentiating metastatic and non-metastatic pNETs; promotes metastasis via MAPK pathway and MMP9 upregulation	135

**Table 7:** Exosomal biomarkers in genitourinary system cancers

Cancer type	Biomarkers	Source	Analytical Technique	Expression	Effectiveness	Reference
	EpCAM, HER2	Serum	Plasmonic metasurface (APM), qRT-PCR, TEM, NTA, SEM, AUROC analysis	Upregulated	AUROC: 99.3% for EpCAM; APM biosensors distinguish BC from controls and classify HER2 levels with P < 0.0001	139
Breast cancer	EpCAM, MUC1	Plasma	CRISPR/Cas12a, Aptamer-chemiluminescence (ACL), TEM, NTA, Western blot, Flow Cytometry	Upregulated	AUROC: EpCAM (+) % = 0.9889, MUC1(+) % = 0.9630; High sensitivity and specificity in differentiating breast cancer from healthy controls	140
	miR-375, PD-L1 mRNA	Serum	Electrochemical analysis, qRT-PCR, TEM, NTA, Western blot	Upregulated	Detection limit: 557 particles/mL for miR-375; Reliable subtype differentiation with homotypic recognition; Quantitative analysis supports diagnosis and monitoring	141
Ovarian cancer	CD81, CD9, EpCAM, EGFR, CD24, CA125	Serum	SERS-Microfluidic platform, TEM, NTA, Western blot, AUROC analysis	Upregulated	AUROC: 0.9467 for early-stage OC (stage I-II); 0.9538 for all OC patients. High sensitivity (1.00) and specificity (0.95) in early diagnosis.	146
	MUC1	Serum	Electrochemical biosensor with EADC, TEM, NTA, SWV	Upregulated	LOD: 30 particles/µL; Linear range: 79 to 315,000 particles/µL; Differentiates OC exosomes with high specificity and sensitivity	147
	miR-1246, miR-141-3p, miR-200a-3p, miR-200b-3p, miR-200c-3p, miR-203a-3p, miR-429	Serum	RNA sequencing, qRT-PCR, TEM, NTA, LASSO regression, AUROC analysis	Upregulated	AUROC: 0.973 (testing cohort), 0.924 (validation cohort); Higher sensitivity and specificity than CA125 for early-stage OC detection	148
Renal cell carcinoma	KMT2D, PREX2	Serum	RNA sequencing, qRT-PCR, Logistic regression, AUROC analysis	Upregulated	AUROC: 0.836 (training cohort), 0.830 (validation cohort) for ccRCC vs. healthy; AUROC: 0.816 for ccRCC vs. benign renal masses	153
	HV1 (Hypervariable Region 1), CYB (Cytochrome B)	Plasma	qPCR, dPCR, NGS, AUROC analysis	Upregulated	AUROC: 0.833 (HV1 long), 0.810 (CYB long); Effective in differentiating RCC cases and assessing aggressiveness	154
	CA9, CD147, CD70	Serum	Western blot, TEM, NTA, IHC, qRT-PCR	Upregulated	Potential to enhance tumor-specific EV isolation; diagnostic markers for ccRCC with high specificity	155

**Table 8:** Exosomal biomarkers in dermal system cancers

Cancer type	Biomarkers	Source	Analytical Technique	Expression	Effectiveness	Reference
	CD63, Cav1	Serum	ELISA, Immuno-magnetic separation, TEM, SEM, DLS	Upregulated	Sensitivity: 87.5% for early-stage melanoma; Enhanced Cav1/CD9 ratio by 7.7-11.3 times compared to bulk exosomes	159
Malignant melanoma	PD1, PD-L1	Plasma	TEM, DLS, NTA, Flow Cytometry	Upregulated	AUROC: 0.86 (PFS, PD1), 0.975 (OS, PD1); independent predictors for resistance to ICI therapy	160
	miR-1180-3p	Plasma	RNA sequencing, qRT-PCR, AUROC analysis, TEM	Downregulated	AUROC: 0.729 for melanoma detection; Associated with tumor proliferation, migration, and invasion	161
Non-melanoma skin cancer	miR-197-5p	Serum	RNA sequencing, qRT-PCR, TEM, NTA	Upregulated	miR-197-5p promotes fibroblast migration and invasion; potential biomarker for MBCC progression and metastasis	166
	circ-CYP24A1	Plasma	RNA sequencing, qRT-PCR, NTA, TEM, Western blot	Upregulated	circ-CYP24A1 promotes cSCC proliferation, migration, and invasion; potential therapeutic target upregulation	167

**Table 9:** Exosomal biomarkers in motor system cancers

Cancer type	Biomarkers	Source	Analytical Technique	Expression	Effectiveness	Reference
	CD63, VIM, EpCAM	Plasma	Microfluidic-SERS, TEM, NTA, Western blot	Upregulated	AUROC: 0.971 (training set); Sensitivity: 100%, Specificity: 90%, Accuracy: 95%	170
Osteosarcoma	THBS1, MS4A1, TCL1A	Plasma	NanoString, qRT-PCR, TEM, NTA	Upregulated	THBS1 associated with poor response to chemotherapy; potential biomarkers for minimal residual disease (MRD)	171
	SENP1 (Sentrin SUMO-Specific Protease 1)	Plasma	ELISA, TEM, NTA, Western Blot	Upregulated	AUROC: 0.90 (1-year DFS), 0.96 (3-year DFS); predictive of poor prognosis and survival outcomes	172

**Table 10:** Clinical trials of cancer exosome biomarkers

NCT number	Biomarkers	Origin	Tumor	Number of included subjects	Status	Start time	Investigators or contacts
NCT06707961	PCA3 mRNA	Abdominal cavity drainage fluid	Prostate cancer	100	Not yet recruiting	2024	Huaqi Zhan *et al.*
NCT05854030	miRNA	Blood	Lung squamous carcinoma	60	Recruiting	2022	Richeng Jiang *et al.*
NCT06342427	miRNA	Blood	Gastric cancer	809	Completed	2023	Ajay Goel *et al.*
NCT03738319	miRNA/ lncRNA	Blood	Epithelia ovarian cancer	160	Unknown status	2018	Lei Li *et al.*
NCT03108677	RNA	Blood	Osteosarcoma	90	Active, not recruiting	2017	Yuhui Shen *et al.*
NCT03874559	Uncertain	Blood	Rectal cancer	30	Recruiting	2018	Andrew Hoover *et al.*
NCT03102268	ncRNA	Bile	Cholangiocarcinoma	80	Unknown status	2017	Lin Miao *et al.*
NCT06342414	exo-miRNA	Blood	Primary liver cancer	400	Recruiting	2024	Ajay Goel *et al.*
NCT04530890	ctDNA	Blood	Breast Cancer	1000	Recruiting	2021	Camille Evrard *et al.*
NCT06278064	Protein	Blood	Upper Gastrointestinal Cancer	562	Recruiting	2024	Li Min *et al.*
NCT04499794	EML4-ALK	Blood	Non-small cell lung cancer	75	Recruiting	2020	Yutao Liu *et al.*
NCT02890849	PD-L1 mRNA	Blood	Non-small cell lung cancer	60	Completed	2016	Jianguo Sun *et al.*
NCT06654622	miRNA	Blood	Colorectal cancer	200	Recruiting	2023	Ajay Goel *et al.*
NCT05270174	lncRNA-ELNAT1	Urine	Bladder cancer	74	Not yet recruiting	2023	Changhao Chen *et al.*
NCT02147418	Protein	Oropharyngeal rinse	Human Papillomavirus-Positive Oropharyngeal Squamous Cell Carcinoma	30	Recruiting	2015	Andrew Cowan *et al.*
NCT03800121	Uncertain	Blood	Sarcoma	34	Recruiting	2018	Alice HERVIEU *et al.*

## Abbreviations

MVBs: multivesicular bodies
ILVs: intraluminal vesicles
DC: dendritic cell
NK: natural killer
TDEs: tumor-derived exosomes
MSC: mesenchymal stem cell
Tregs: regulatory T cells
MDSCs: myeloid-derived suppressor cells
LOX: lysyl oxidase
EMT: epithelial-mesenchymal transition
ABC: ATP-binding cassette
BBB: blood-brain barrier
NSCLC: non-small cell lung cancer
SCLC: small cell lung cancer
ICDSE: Integrated Concentration and Determination System of Exosomes
PFS: progression-free survival
OS: overall survival
sEVs: small extracellular vesicles
TAAs: tumor-associated antigens
SERS: Surface-enhanced Raman Spectroscopy
AI: Artificial Intelligence
NPC: nasopharyngeal carcinoma
EBV: Epstein-Barr virus
LSCC: laryngeal squamous cell carcinoma
AUC: Area Under the Curve
GC: gastric cancer
HCC: hepatocellular carcinoma
PDAC: pancreatic ductal adenocarcinoma
CRC: colorectal cancer
ALL: acute lymphoblastic leukemia
AML: acute myeloid leukemia
CLL: chronic lymphocytic leukemia
CML: chronic myeloid leukemia
MM: multiple myeloma
HL: Hodgkin lymphoma
NHL: non-Hodgkin lymphoma
PAs: pituitary adenomas
PCNSL: primary central nervous system lymphoma
PTC: papillary thyroid cancer
MTC: medullary thyroid cancer
FTC: follicular thyroid cancer
ATC: anaplastic thyroid cancer
PNETs: pancreatic neuroendocrine tumors
BC: breast cancer
OC: ovarian cancer
RCC: renal cell carcinoma
MM: malignant melanoma
NMSC: non-melanoma skin cancer
RMS: rhabdomyosarcoma
